# Sound asleep: sensory decoupling during sleep depends on an infant’s sensory profile

**DOI:** 10.1093/sleep/zsag010

**Published:** 2026-01-20

**Authors:** Anna De Laet, Morgan Whitworth, Hope Fincham, Alpar S Lazar, Rachael Bedford, Teodora Gliga

**Affiliations:** School of Psychology, University of East Anglia, Norwich, United Kingdom; School of Psychology, University of East Anglia, Norwich, United Kingdom; School of Psychology, University of East Anglia, Norwich, United Kingdom; School of Psychology, University of East Anglia, Norwich, United Kingdom; School of Biological and Behavioural Sciences, Queen Mary University of London, London, United Kingdom; School of Psychology, University of East Anglia, Norwich, United Kingdom

**Keywords:** infancy, sensory processing, EEG, auditory stimulation, sleep spindles, slow waves, nap

## Abstract

Initiating and maintaining sleep requires gating of sensory input. Sensory processing differences, such as elevated sensory reactivity, have emerged as a potential driver of sleep difficulties in autism. Both sensory and sleep difficulties are prevalent in autistic individuals and emerge early in development. Here, we use polysomnography to understand how infant sensory reactivity affects the ability to maintain sleep in a quiet or noisy environment. Forty-four 8- to 11-month-old infants at typical and elevated likelihood for autism participated in a lab-based nap study consisting of two counterbalanced visits, a baseline and an auditory stimulation condition. In the stimulation condition, 60 dB pure tones were played during sleep. We measured slow waves and sleep spindles, electroencephalogram features previously linked to the ability to protect sleep from sensory disturbance. We show that higher caregiver-reported sensory reactivity was significantly associated with lower slow wave activity and density, across both nap conditions. In the stimulation condition, infants with elevated sensory reactivity had even further decreased slow wave density and lower sleep spindle density. Comparisons of pre- and poststimulus windows showed that, rather than triggering immediate event-related disruptions, auditory input and sensory reactivity alter sleep microstructure across the longer timescale of the entire nap. Thus, highly reactive infants experience disruptions in their ability to enter or maintain periods of sensory disconnection, accentuated by the presence of auditory noise.

## Graphical Abstract

**Graphical Abstract:**
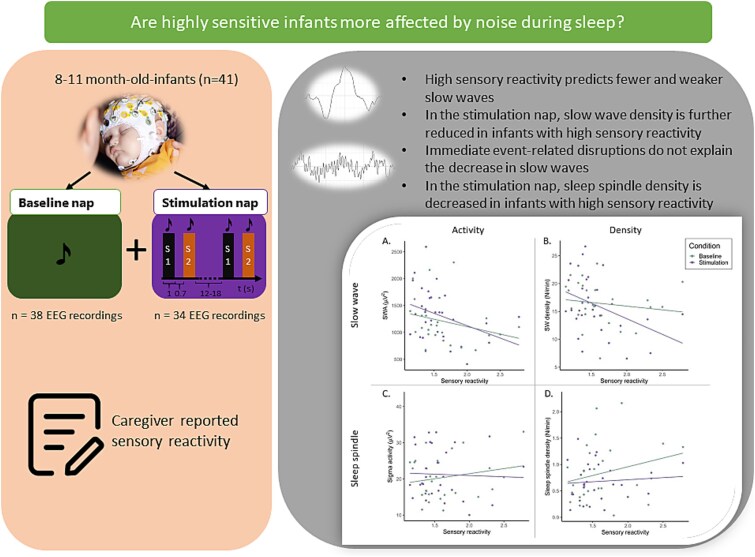

Statement of Significance Good sleep relies on a (at least partial) disconnection from the sensory environment and the ability to gate irrelevant sensory input. Infants who tend to be highly reactive to sensory input during wakefulness, a common early emerging symptom of autism, often also have sleep difficulties. Here we inquired whether sleep difficulties have a sensory origin by experimentally testing the effects of auditory input on the sleeping brain of infants with a variety of sensory profiles. We show that infants with high sensory reactivity experience disruptions in their ability to enter or maintain periods of sensory disconnection, accentuated by the presence of auditory noise.

## Introduction

Sleep is important at every stage of life, but may be especially important during early development because of the role it plays in regulating cortical plasticity [1, 2]. Early sleep predicts behavioural outcomes and mental health later in life [3, 4] and sleep difficulties are prevalent in neurodevelopmental conditions [5–7]. As early as the first year of life, infants at an elevated likelihood (EL) of developing autism–—because they have an older autistic sibling—take longer to fall asleep and wake up more frequently than peers at typical likelihood (TL) for autism [8–10].

There is an emerging understanding that sensory processing differences, a core diagnostic feature of autism, may contribute to sleep difficulties [11]. Sensory differences are a core diagnostic feature of autism, but also common in other neurodevelopmental conditions [12], and include phenotypic manifestations such as heightened (hyperreactive) and diminished (hyporeactive) responses to everyday sensory stimuli. They are among the earliest traits of autism predictive of later diagnosis [13, 14]. Sensory processing differences associate with sleep difficulties in autistic adults and children (see review by Lane et al. [15]), as well as in nonautistic individuals [16], and are evident as early as the first year of life [9]. One possibility is that sensory processing differences lead to sleep difficulties via ineffective gating of sensory input during sleep. Effective gating of sensory input is critical to establish and maintain sleep [17, 18]. Certain features of sleep electroencephalograms (EEG) or sleep microarchitecture are associated with sensory “decoupling” from the environment [19]. In particular, hallmark oscillations of non-rapid eye movement (NREM) sleep, such as slow waves (large-amplitude oscillations in the 0.5–4 Hz range) and sleep spindles (waxing and waning phasic oscillations in the 9–16 Hz range), are thought to protect the sleeper from incoming sensory input [20–22]. Active upregulation of slow waves and spindles was documented in response to sensory input [23–25]. These measures of sensory decoupling are altered in autistic individuals. Reduced sleep spindle density [26–28] and decreased slow wave activity have been reported in autistic children and adolescents relative to nonautistic individuals [29, 30] (but see studies [31, 32]). Autistic children aged 6–13 years also have fewer K-complexes, a distinct type of slow wave in sleep stage N2 [33]. K-complexes are thought to reflect a cortical response to sensory input, while simultaneously contributing to sleep protection by preventing stimulus-induced awakenings [34, 35].

A recent study in 10-month-old infants at typical and elevated likelihood for autism showed that a neural measure of sensory gating, measured as the suppression of repeated tactile input during wakefulness, is associated with prolonged sleep onset [9], suggesting that poor sensory gating interferes with the establishment of sleep. The current study aimed to investigate how sensory reactivity affects sleep architecture, by capturing neural measures of sensory gating during sleep. First, we ascertained whether alterations in sleep microarchitecture relate to phenotypic measures of sensory differences. Second, we aimed to test whether gating of sensory input is responsible for sleep disturbance in infants with high sensory reactivity, by experimentally manipulating sensory input, during sleep.

To address these aims, we measured the impact of auditory input during a nap, in 8- to 10-month-old infants, a key age at which sleep differences emerge between EL and TL infants [9]. We captured sleep macroarchitecture and microarchitecture using EEG and measured sensory reactivity through caregiver report. The latter captures an infant’s typical affective and behavioural responses to sensory input [36] by combining a range of items across sensory modalities (e.g., “My child startles easily at sound compared to same-age children”). If poor gating of sensory input causes sleep difficulties in highly reactive infants, we expect markers of sensory decoupling to be affected, i.e., lower slow waves or spindles, particularly when sleep is challenged by sensory input.

## Methods

The analysis of this study was preregistered on the Open Science Framework (osf.io/xkpz9). Deviations from the preregistration are described in Table S1.

### Participants

Forty-four infants, aged 8–11 months, participated in a nap study, consisting of two visits (baseline and stimulation condition, counterbalanced) in the Sleep and Brain Research Unit at the University of East Anglia, United Kingdom. Three infants did not fall asleep at the initial visit and did not return for subsequent attempts, leaving 41 infants (female = 18) who contributed at least one nap. One infant failed to nap initially, but returned for two successful naps. Thirty-one infants completed both visits. Data from infants who completed at least one visit were included in the analysis, resulting in a total of 72 nap recordings (baseline = 38, stimulation = 34). Sample sizes vary depending on the measures used, and the exact sample size for each model is provided in the results table. To ensure infants in the study sample had a variety of sensory profiles, the study recruited infants with an older sibling with or without an autism diagnosis, as sensory processing differences are prevalent in the majority of autistic people, estimated up to 90% [37, 38]. Infants were classified as being at elevated likelihood for autism (EL; *n* = 8, female = 3) if they had an older sibling diagnosed with autism (*n* = 7) or undergoing diagnosis (*n* = 1), based on parental report. The autism traits of the older siblings were also assessed using the Social Communication Questionnaire (mean score = 19, SD = 8.5) [39]. Five siblings scored above the cutoff score (≥11; [40, 41]); two siblings scored below the cutoff (scores of 9 and 10) despite having an autism diagnosis and an SCQ score was missing for one sibling. Infants without an autistic first-degree relative were considered at typical likelihood for autism (TL; *n* = 33, female = 15). Within the participant sample, a pair of monozygotic twins (TLs) and a pair of siblings (ELs) took part in the study. Participants were recruited on a voluntary basis through the participant database of the University of East Anglia and ads on social media. Additionally, organizations and autism charities were contacted to advertise the study. Participants received a £10 shopping voucher for each visit and reimbursement for travel and accommodation if needed. In addition, they received a book at the first visit and a T-shirt at the second visit. Written consent by the caregiver was acquired before the onset of the study. Ethical approval was given by the Ethics Committee of the School of Psychology of the University of East Anglia (ethics reference code: ETH2324-0296).

### Experimental protocol

Families arrived at the lab just before the infant’s usual naptime and returned at the same time on their second visit. When comparing the lights-off time between visits, 74% of infants who completed both visits had less than 1 hour difference across visits (median difference = 19 minutes, range = 1–203 minutes). Most infants took part in a morning visit, between 8 am and 1 pm. Five infants visited the lab in the afternoon, between 1 and 5 pm. No significant differences on micro- and macroarchitecture were found between morning and afternoon naps (see online supplementary materials and Table S2). The average time between visits was 15 days (SD = 14 days; range 2–61 days). Caregivers were encouraged to settle their infant as they typically would at home. Infants either slept in the caregiver’s arms on an armchair (*n* = 29), or separate from the caregiver in a cot or on a bed (*n* = 12). These sleeping arrangements in the sleep lab were consistent across the two visits for each infant. The sleeping arrangement did not significantly affect any micro- or macroarchitecture variables, except sleep stage distributions (see online supplementary materials and Table S3). When possible, caregivers filled out questionnaires during the nap; otherwise they were completed at home. An experimenter stayed in the room with the caregiver and infant, making notes during the session. Caregivers were encouraged to let their child nap until they woke up naturally. However, on four occasions caregivers woke the infant up prematurely for practical reasons (e.g., picking up the older sibling from the nursery). These sessions were excluded from the analysis of sleep duration.

**Figure 1:**
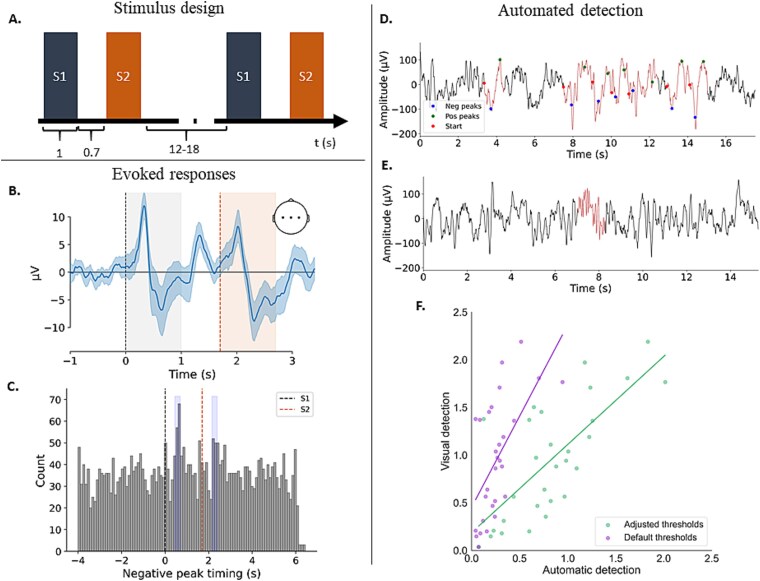
(A) Stimulus design. Time is represented in seconds. (B) Evoked responses averaged across the central channels across N2 and N3 (*n* = 28). (C) Counts of the negative peaks of slow waves are time-locked to S1 and S2. Clear increases in detected SWs are seen in the 450–700 ms window after stimulus onset (highlighted in blue). In artifact-free N2 segments from 32 nap recordings, 3571 slow waves were detected. On average the recordings had 41 pairs of stimuli in artifact-free N2. (D) A representative example of automatically detected slow waves in N3. (E) A representative example of an automatically detected sleep spindle in N2. (F) Associations between visually and automatically detected sleep spindles using the default parameters (purple) and the optimized parameters (green). Data from the training dataset are shown.

Infants napped in two different conditions: *baseline* and *stimulation*. The order of conditions was randomly counterbalanced across the study (see Table 1). In the stimulation condition, auditory stimulation was played from two speakers that were positioned approximately 50 cm from the child’s head. An experimenter was always in the room with the parent and infant, monitoring the recording. The experimenter started the stimulation once the infant was asleep for a couple of minutes, as confirmed by the caregiver and clearly visible on the EEG signal. The stimulation was only stopped when infants awoke from the nap and showed no sign of falling back asleep. Auditory input was applied in the form of 60 dBA (measured at the infant’s head) 225 Hz pure tones, lasting 1 s each and presented in pairs (henceforth S1 and S2; Figure 1, A). The volume was set at 60 dB to ensure this was below the arousal threshold while still being noticeable. Previous studies in infants show that the arousal threshold for auditory stimuli tends to be between 70 and 100+ dB [42, 43]. Keeping the volume constant across participants, rather than adjusting it to individual arousal levels, was part of the design to avoid obscuring variability due to differences in sensory reactivity. The intrastimulus interval was 700 ms and the interval between pairs varied randomly between 12 and 18 seconds. This interstimulus interval and jitter were chosen to minimize habituation to and anticipation of the stimulus. The total number of pairs of stimuli depended on the nap length. On average 175 pairs of stimuli were presented, ranging from 89 to 325.

**Table 1:** Descriptive statistics per nap condition

	Baseline nap	Stimulation nap
*n*	38	34
M:F	21:17	17:17
EL:TL	7:31	7:27
Visit 1:visit 2	22:16	19:15
Age (in days)	303 (34)	300 (32)
**Sleep macroarchitecture**		
Sleep onset latency (minutes)	10.3 (6.6)	11.5 (7.2)
Nap duration (minutes)	62.2 (23.5)	50.9 (16.7)
Wake after sleep onset (minutes)	0.9 (1.6)	0.6 (1.6)
Sleep efficiency (%)	96.9 (3.7)	97.5 (3.1)
Sleep stages (minutes)		
N1	12.1 (9.7)	10.0 (4.6)
N2	21.5 (11.4)	17.1 (10.4)
N3	20.8 (9.1)	20.8 (8.4)
REM	5.8 (6.7)	4.1 (5.9)

Sleep onset latency was measured as the time from the lights off moment to the start of the first epoch of sleep (including N1). Sleep efficiency is calculated as the total sleep time (N1 + N2 + N3 + REM) divided by the duration from the first to last period of sleep. Mean (SD). M = male; F = female.

### E‌EG data acquisition

Brain activity was recorded using a wearable LiveAmp with 32 channels (Brain Products GmbH, Gilching, Germany) placed within an EEG sensor cap according to the 10–20 system. Different cap sizes were available, sized to the infant’s head circumference (actiCap snap, Brain Products GmbH, Gilching, Germany). Four out of thirty-two electrodes (FT9, P7, P8, and FT10) were used to measure electro-oculography (EOG) and electromyography (EMG). The EEG cap was turned inside out and all the electrodes were pre-gelled to minimize the capping time, and caregivers were briefed about the study. An experimenter applied the cap while the infant was distracted. The EOG and EMG electrodes were then applied on the face using stickers. The whole procedure lasted around 5 minutes. The sampling rate during data acquisition was 500 Hz and the online reference was FCz.

### E‌EG pre-processing and sleep staging

All the pre-processing steps were performed in MNE Python [44]. Data were filtered between 0.2 and 40 Hz using a first-order Butterworth bandpass filter. Notch filters at 50, 100, 150, and 200 Hz were added to exclude line noise. Data were re-referenced offline to the contralateral mastoids for sleep staging and visual arousal detection. Manual sleep staging was performed using the graphical user interface Sleep from Visbrain, a Python-based package [45, 46]. Sleep staging was done on 30 second epochs by two individual scorers based on the AASM criteria and guidelines from the Pediatric Task Force [47, 48]. All the hypnograms were compared between the two scorers. Where the hypnograms deviated from each other, the scorers discussed them until an agreement was reached. Visually detected arousals—of 3 s minimally—were marked on the hypnograms. Arousal detection was based on guidelines from the International Paediatric Work Group [49] and The Pediatric Task Force [47], but slightly adapted due to the absence of equipment measuring heart rate or breathing patterns. An arousal was marked when two of the following criteria were present: (1) a gross body movement; (2) an increase in chin EMG amplitude (unless associated with sucking); and (3) an abrupt change in the EEG background frequency (of at least 1 Hz) for a minimum of 3 seconds. Arousal detection in three recordings was compared and discussed by two raters (ADL and HF), and 100% agreement was reached on event level. Subsequent arousal detection was done independently by each rater. On average, 5.4 arousals (4.0 SD) were detected per nap. Summary statistics of arousals are also provided per sleep stage and per condition in Table S4. After arousal detection, automatic artifact rejection was performed in all sleep stages using the artefact rejection function from YASA on 1 second windows [50]. On average, 2.1 minutes of a nap (SD = 1.3) were identified as artifacts and excluded from analyses, equivalent to 3.6%. The percentage of artifacts was not significantly different between the baseline (mean = 3.7, SD = 1.7) and stimulation (mean = 3.4, SD = 1.9) conditions (*t* = 0.67, *p* = .51). The mean durations and percentages of artifacts per sleep stage and condition are presented in Table S5. Arousal density was measured as the total number of arousals divided by the total sleep period (sleep onset − sleep offset) excluding arousal time. Arousals were excluded if they were caused by a known external factor other than the auditory stimulation, such as the caregiver moving the baby. These events were observed and noted down during testing sessions by an experimenter in the room. Arousal density was not calculated in 10 recordings, due to bad-quality chin electrodes, which prevented the reliable detection of arousals. Arousals and artifacts were excluded from further analyses to avoid confounders. Additionally, data were re-referenced to the linked mastoids for any further analysis. As pre-registered, recordings which had one or two bad-quality mastoid channels were excluded (*n* = 6). Although alternative reference methods, such as an average reference or contralateral mastoid reference, could retain more data, reliable average referencing requires a high-density electrode montage [51] and varying reference choices can affect spindle amplitudes (and therefore spindle detection) and topographical patterns. Consistent use of linked mastoids thus ensured interpretability across recordings, which was prioritized over sample size.

### Sleep microarchitecture

#### Slow waves

Slow waves were captured in two distinct ways: as the average activity in the slow-wave frequency band and as discrete events. Additionally, evoked K-complexes were measured time-locked to the stimulation.

Slow wave activity (SWA; 0.5–2 Hz) was measured in sleep stages N2 and N3 in electrodes F3, Fz, F4, FC1, FC2, C3, Cz, C4, O1, and Oz. This region of interest was based on SWA patterns in a similar age group [52] and was confirmed in a subsample of recordings as detailed in the pre-registration. The absolute power spectral density was calculated using Welch’s method and a Hann window with a length of 4 s. The median was used when averaging periodograms. Next, SWA was averaged across the electrodes, using the median.

Discrete slow wave events were detected using the automated slow wave detection algorithm of the YASA package. A representative example of automatically detected slow waves in sleep stage N3 is shown in Figure 1, D. The default settings were used for the frequency band (0.5–2 Hz), negative peak duration (0.3–1.5 seconds), and positive peak duration (0.1–1 seconds). Due to the higher amplitude of infant slow waves relative to adults (Frey et al. 2016), the default upper amplitude limits were removed, and only the lower thresholds were applied: greater than 40 μV for the negative peaks, greater than 10 μV for positive peaks, and greater than 75 μV for the peak-to-peak amplitude. Slow wave density was calculated as the number of detected SWs in artifact-free N2 and N3 divided by the time in N2 and N3 in the same ROI as the SWA. The median SW density was then taken across these electrodes, resulting in one value for SW density per participant per nap.

Given that central areas exhibit the largest K-complex amplitudes in children [53], K-complexes were detected in channel Cz. Detection was performed on 16-second-long artifact-free N2 segments, starting at −4.15 seconds and ending at 12.35 seconds time-locked to S1. This length was chosen to meet the minimum length requirement for the algorithm to detect SWs. In adults, K-complexes, evoked by sensory input, appear in a reliable and well-defined time window. Specifically, the negative peak of an evoked K-complex occurs between 450 and 700 ms after stimulus onset [54]. However, it is unclear whether these timings are the same in infants. To determine this, we plotted the timings of the negative peaks of automatically detected SWs relative to stimulus onset. As shown in Figure 1, B and C, there was a clear increase in the production of SWs following S1 and S2 in the expected time window. We therefore proceeded to quantify the evoked K-complexes by counting the number of SWs with a negative peak in the 450–700 ms time window—called “the stimulus ON window”—in artifact-free N2 segments. To account for differences in N2 length between nap recordings, the total number was divided by the number of stimuli, resulting in the K-complex likelihood. Because of a refractory period after an evoked K-complex [55], only the responses to S1 were considered, but not those to S2.

These counts likely reflect a mix of both evoked and spontaneous K-complexes. To account for this, we included a control condition by comparing counts in a prestimulus window (stimulus OFF) of equal length, ranging from −1250 to −1000 ms relative to S1 onset (see time-locked analyses below).

#### Sleep spindles

Similarly to SWs, sleep spindles were captured in two distinct ways: (1) as the average activity in the sigma frequency band and (2) as discrete events. Additionally, evoked sleep spindles were measured time-locked to the stimulation. Sigma power (9–16 Hz) was calculated in artifact-free N2 in channels F3, Fz, F4, FC1, FC2, C3, Cz, and C4. This ROI is based on Sokoloff et al. [52] and was confirmed in a subsample of recordings in the pre-registration. Absolute power was calculated using Welch’s method and a Hann window with a length of 4 s. Median sigma activity was calculated across the electrodes in the ROI.

### Sleep spindle detection

Sleep spindles were detected using YASA’s automated spindle detection function [50]. The algorithm has parameters and thresholds that can be adjusted. The default settings of YASA are based on sleep recordings of 2–80-year-olds (not including the age range of this study). Sleep spindles undergo significant developmental changes, especially in the first year of life, and we therefore modified some parameters based on previous infant literature. Given that infant sleep spindles often have lower amplitudes [56, 57] and longer durations, and do not have their typical waxing and waning shape, sleep spindle duration was set to 0.5–4 seconds and the minimum distance between sleep spindles to 750 ms. We also opted for a wider frequency range (9–16 Hz) to maximize the inclusion of sleep spindles [58, 59].

Additionally, the YASA algorithm detects sleep spindles based on three thresholds, which largely determine the sensitivity of the algorithm: the root mean square (RMS), the relative power, and the moving correlation. For more information on these thresholds, see the YASA website. Using the default threshold settings, the algorithm did not perform as well on this dataset (*F*_1_ score = 0.4) as on adult recordings (*F*_1_ score = 0.7) [60]. The default settings gave particularly bad recall scores—meaning they missed many sleep spindles but had good precision scores. The sleep spindles they detected tended to be true sleep spindles (see Table S6). We therefore systematically adjusted these thresholds to optimize the algorithm performance on this particular dataset. Using common principles for algorithm validation, we optimized threshold settings based on a training dataset (a random selection of 29 recordings) to maximize agreement with a ground truth—here the manually annotated sleep spindles in artifact-free N2 and N3 in channel C4. Performance was evaluated based on *F*_1_ scores, the weighted mean of precision, and recall scores [61, 62]. The best-performing adjusted algorithm was then tested on a separate validation dataset (*n* = 29) and should perform similarly to the training dataset.

### Algorithm adaptation

See the supplemental materials for a full description of the approach and results. Python scripts are available on the Open Science Framework (osf.io/3ujr4).

The best-performing algorithm in the training datasets had an *F*_1_ score of 0.59 using thresholds set to the following: RMS 85% = 1.275, correlation 80% = 0.52, and relative power 65% = 0.13. These threshold settings were used on the validation set and yielded similar performance metrics (*F*_1_ = 0.55; see Table S6).

As *F*_1_ scores are dependent on the number of detected spindles, we also tested whether the best-performing algorithm showed an improvement compared to the default algorithm in the estimated sleep spindle density (number per minute), a measure that adjusts for sleep recording length. Sleep spindle densities were calculated for the default algorithm, the best-performing adjusted algorithm, and manual detection. Pearson correlations between manual detection and both the default and adjusted algorithms were significant (*r* = 0.61, *p* < .001; *r* = 0.73, *p* < .001, respectively). The correlation with the adjusted algorithm was significantly higher than the correlation with the default algorithm (*z* = −1.97, *p* = .049), supporting the choice for the adjusted algorithm (see Figure 1, F). Figure 1, E shows a representative example of an automatically detected sleep spindle with the adjusted thresholds.

Sleep spindle density was calculated as the number of sleep spindles in artifact-free N2 divided by the time in N2 and averaged across the ROI (F3, Fz, F4, FC1, FC2, C3, Cz, and C4).

To test whether sleep spindles were evoked by stimulation, sleep spindles were counted immediately after the stimulus presentation. While the adult literature defines the timing of evoked K-complexes, there is less consensus about the timing—or even the existence—of evoked sleep spindles [25, 63, 64]. When evoked sleep spindles have been reported, the majority of them started around 2 seconds after stimulus onset [23]. Because evoked responses in infant EEG tend to be delayed compared to adults [65], we opted for a longer poststimulus time window in our analyses. Sleep spindles that started within 0–3.4 seconds of S1 onset—referred to as the “ON window”—were classified as in response to the stimulation. This window included S1 (1 second), the S1–S2 interval (700 ms), S2 (1 second), and a post-S2 window of the same length as S1 (700 ms). Due to the stimulus design, the maximum window length was constrained to 3.4 seconds to ensure S1 and S2 were included and poststimulus intervals of equal lengths for S1 and S2. Exploratory time frequency plots up to 5 seconds post S1 onset show no significant clusters of altered activity in the sigma range beyond 3.4 seconds (see Figure S1), suggesting no large average evoked responses were missed due to the window choice. Sleep spindles that started in the 3.4 seconds before S1 onset (−3.4 to 0)—called the OFF window—were considered the control condition. For these analyses, artifact-free N2 segments of 8.8 seconds were selected, ranging from −4.4 to +4.4 relative to S1 onset. This allowed for a buffer zone of 1 second at each end of the segment to avoid interference of edge artifacts with sleep spindle detection. From these counts, the sleep spindle likelihood was calculated by dividing the total number of sleep spindles per recording by the number of stimuli. On average there were 48 stimuli per recording in artifact-free N2. The number ranged from 14 to 108. For these analyses, sleep spindles were automatically detected in channel Cz.

### Sensory reactivity

Sensory processing differences were measured with the Sensory Profile 2 (SP-2 [66]) questionnaire for ages 7–36 months. Caregivers indicate the frequency of their child’s behaviour on a five-point scale ranging from almost always to almost never. The SP-2 groups items into four quadrants: low registration, sensation seeking, sensory sensitivity, and sensation avoidance. For this study, items from the sensory sensitivity (13 items, e.g., “My child startles easily at sound compared to same-age children”) and sensation avoidance quadrant (11 items, e.g. “My child withdraws from unexpected touch”) were combined and averaged to obtain one composite score per participant for which we use the term *sensory reactivity*. Both quadrants contain questions from the general, auditory, oral, touch, and behavioural modality. Sensory sensitivity additionally consists of one question from the movement modality. These two quadrants are chosen because they are consistently correlated with sleep difficulties [67], including in infants [9]. Items were reverse-scored, so high scores represent greater affective and behavioural reactivity to sensory input. Items on the combined quadrants demonstrated good internal consistency with a Cronbach’s alpha of 0.85. On the SP-2, caregivers have the option to respond with “not applicable,” in contrast to the first version of the questionnaire. Sensory reactivity was calculated by taking the average score of all the questions not marked as “not applicable.” Forty-nine percent of caregivers completed all the questions of the sensory reactivity subscale. However, 20% of the participants answered “not applicable” to more than 25% of the questions. The high frequencies of “not applicable” likely reflect a high variability in developmental milestones in 8–11-month-olds and the inappropriateness of some questions for infants at the lower end of the age range covered by the SP-2 (7–36-month-olds). For example, one of the questions that were answered “non-applicable” by 23% of the responders was “My child prefers one texture of food (for example, smooth, crunchy).” It is possible that not all the infants were having a variety of solid foods yet at 8 months, when invited to our study. Whether the number of nonapplicable responses falls within the typical range is uncertain, as to our knowledge no studies have reported this missingness in their samples. Out of 24 questions, respondents had a median of 1 not applicable response (min = 0, max = 19). The rate of missing responses was not related to the average scores (*r* = −0.2, *p* = .22) and therefore no participants were removed based on the amount of not-applicable answers.

### Statistical analysis

As a first step, the distribution of the variables was checked for the presence of outliers. Any outlier above 4 SD from the mean was Winsorized to one unit above the highest nonoutlier value. Values between 3 and 4 SD were visually checked on a scatterplot in relation to other variables. As a result, two values for sleep spindle density in N2 (1 EL and 1 TL) and one value for arousal density (TL) were Winsorized. To ensure results were not affected by this practice, a robustness check was run by removing outliers for the relevant analyses, which is included in the supplemental materials (Table S7).

Linear mixed effect models (LMMs) were used to account for the within-person variance using the R package lmerTest [68]. Parameter estimates were obtained by maximum likelihood estimation, and degrees of freedom and *p*-values for fixed effects were derived using the Satterthwaite approximation, which has been shown to be more robust for a small sample [69].

LMMs were run separately for all the outcome variables of interest: sleep macro-architecture variables (nap duration and sleep stage distribution), arousal density, SWA, slow wave density, sigma activity, sleep spindle density, and sleep spindle and K-complex likelihood. Studies often also report on other spindle characteristics such as sleep spindle duration, frequency, and absolute power; the results for these can be found in the supplemental materials (Table S8). In all the models, participants were added as a random intercept, and stimulation, sensory reactivity, sex, and age as fixed effects. Depending on the model, the variable stimulation was defined either as the nap condition (baseline = 0 vs stimulation = 1) or as the stimulus window (OFF = 0 vs ON = 1). Stimulus window was used when comparing event occurrence—sleep spindle and K-complex likelihood—before and after the stimuli (OFF vs ON windows) within the stimulation naps only.

#### Equation 1: mixed effect model

The base model includes the main effects (a), and the interaction model includes the interaction term (b). *y* = sleep variable of interest.


(1a) \begin{equation*} y\sim \mathrm{stimulation}+\mathrm{sensory}\ \mathrm{reactivity}+\mathrm{sex}+\mathrm{age}+\left(1|\mathrm{subject}\right) \end{equation*}

(1b) \begin{eqnarray*} y\sim \mathrm{stimulation}+\mathrm{sensory}\ \mathrm{reactivity}+\mathrm{sex}+\mathrm{age}\nonumber\\+ \mathrm{condition}\ast \mathrm{sensory}\ \mathrm{reactivity}+\left(1|\mathrm{subject}\right) \end{eqnarray*}

First the models were run with the main effects only (Equation 1a); then the interaction effect between stimulation and sensory reactivity was added (Equation 1b). Estimates and significance values for the main effects were taken from the first model, while those for the interaction effect were taken from the second. As a sensitivity check, autism likelihood status was added as a control variable to the models (see equations 2a and 2b). Although not all the continuous outcome variables were normally distributed, LMMs are fairly robust to non-normality (Schielzeth et al., 2020).

#### Equation 2: sensitivity analyses

Autism likelihood status is added to the base model (a) as well as the interaction model (b).


(2a) \begin{eqnarray*}&& y\sim \mathrm{stimulation}+\mathrm{sensory}\ \mathrm{reactivity}+\mathrm{sex}+\mathrm{age}\nonumber\\&&+\mathrm{autism}\ \mathrm{likelihood}+\left(1|\mathrm{subject}\right) \end{eqnarray*}

.


(2b) \begin{eqnarray*}&& y\sim \mathrm{stimulation}+\mathrm{sensory}\ \mathrm{reactivity}+\mathrm{sex}+\mathrm{age}\nonumber\\&&+\mathrm{autism}\ \mathrm{likelihood}+\mathrm{condition} \ast \mathrm{sensory}\ \mathrm{reactivity}\nonumber\\&&+\left(1|\mathrm{subject}\right) \end{eqnarray*}

## Results

### Effects of stimulation on sleep macroarchitecture

**Figure 2:**
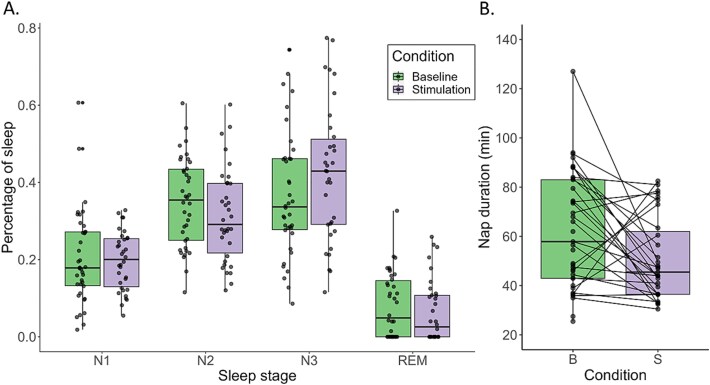
Sleep macroarchitecture in the baseline (green) and stimulation (purple) conditions. (A) Differences in sleep stage distributions are not significant between conditions in the full model (see results in the main text). (B) Sleep duration is significantly shorter in the stimulation condition.

Sleep onset times in the sleep lab (see Table 1) were comparable to typical nap onset times reported by caregivers in sleep diaries a week prior to the sleep lab visit (11.7 ± 5.7 minutes), suggesting sleep onset was similar despite the unfamiliar environment and wearing an EEG cap. On average, infants slept 60.2 ± 23.5 minutes in the baseline condition. In 4- to 5-month-olds, Ventura et al. [70] report a mean sleep cycle length of 42.8 minutes in daytime naps, suggesting infants in the current study completed 1–2 sleep cycles. Auditory stimulation significantly affected the duration of the nap (Est. = −12.2, *p* = .018), with shorter nap durations in the stimulation condition (50.9 ± 16.7 minutes) compared to the baseline (62.2 ± 23.5) (Figure 2, B). Nap duration was not significantly associated with sensory reactivity (Est. = -0.4, *p* = .949), nor was there a significant interaction between sensory reactivity and nap condition (Est. = 7.1, *p* = .577).

A generalized LMM with a zero-inflated beta regression and logit link function was run to assess the effects of condition and sensory reactivity on the distribution of sleep stages. As expected, different sleep stages took up varying proportions of the nap (reference category N1; N2: Est. = 0.70, *p* < .001; N3: Est. = 0.91, *p* < .001; REM: Est. = −0.39, *p* = .007). The distribution of sleep stages did not significantly differ between the baseline and stimulation nap (Cond.xN2: Est. = −0.20, *p* = .35; Cond.xN3: Est. = 0.17, *p* = .44; Cond.xREM: Est. = −0.13, *p* = .65), nor did sensory reactivity moderate this effect (CondxN2xsens: Est. = 0.85, *p* = .12; CondxN3xsens: Est. = −0.70, *p* = .21; CondxREMxsens: Est. = −0.71, *p* = .39), indicating that time spent in each sleep stage did not vary across nap conditions depending on an infant’s sensory profile.

### Higher sensory reactivity predicts fewer and weaker slow waves

**Figure 3:**
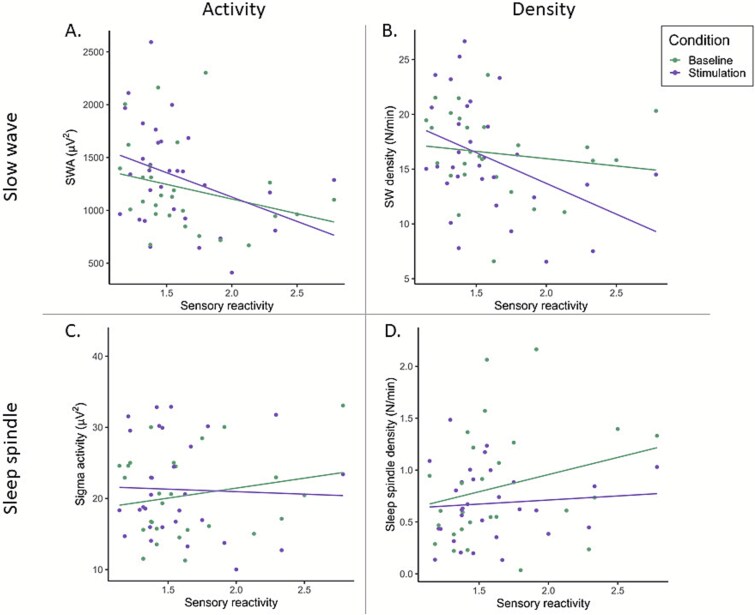
Relationship between sleep microarchitecture measures and sensory reactivity scores in the baseline (green) and stimulation (purple) conditions. (A) Slow wave activity. There is a significant main effect of sensory reactivity on slow wave activity, but no significant interaction effect. (B) Slow wave density. There is a significant main effect of sensory reactivity on slow wave density and a significant interaction effect of sensory reactivity and nap condition on slow wave density. (C) Sigma activity. There is no significant main effect or interaction effect of sensory reactivity on sigma activity. (D) Sleep spindle density. There is a significant interaction effect of sensory reactivity and nap condition on sleep spindle density, but no main effect.

We measured both discrete slow wave events as well as slow wave activity (0.5–2 Hz) to strengthen the robustness of our findings and to better understand mechanisms of sensory interference during sleep. Slow wave activity (SWA) was not significantly different between the baseline and stimulation nap (Est. = −130, *p* = .138) but higher scores on sensory reactivity associated with lower SWA (Est. = −382, *p* = .033; see Figure 3, A). However, there was no significant interaction effect between sensory reactivity and condition (Est. = −165, *p* = .45), indicating that infants who score higher on sensory reactivity tend to have lower SWA, regardless of the sensory environment. Nap condition did not have a significant effect on SW density (Est. = −0.47, *p* = .598). Similarly to SWA, higher sensory reactivity scores predicted lower SW density (Est. = −3.67, *p* = .033; see Figure 3, B). In contrast to SWA, there was also a significant interaction effect of sensory reactivity and condition on SW density (Est. = −4.34, *p* = .046). The SW density of infants with high sensory reactivity scores was decreased in the stimulation condition, but not that of infants with lower sensory reactivity scores. See the results summarized in Table 2.

**Table 2:** Results from the mixed effect models of sleep microarchitecture

	Standardized β	95% CI	Unstandardized β	SE	*t* or *z*	*P*-value
*Y* = arousal density 56 nap recordings, 31 participants						
Condition (B vs S)	0.04	−0.32 to 0.39	0.002	0.011	0.208	.837
Sensory reactivity	−0.02	−0.35 to 0.32	−0.003	0.028	−0.094	.926
Sex (0 = M, 1 = F)	0.02	−0.68 to 0.72	0.001	0.022	0.051	.959
Age	−0.04	−0.34 to 0.26	0.000	0.000	−0.283	.779
Sleep arrangement	−0.36	−0.95 to 0.24	−0.023	0.019	−1.205	.234
Sensory reactivity* cond.	−0.04	−0.40 to 0.32	−0.007	0.030	−0.225	.824
*Y* = SW density 58 nap recordings, 35 participants						
Condition (B vs S)	−0.10	−0.49 to 0.28	−0.473	0.886	−0.534	.598
Sensory reactivity	−0.32	−0.60 to −0.03	−3.672	1.649	−2.227	**.033**
Sex (0 = M, 1 = F)	−0.46	−1.04 to 0.11	−2.118	1.317	−1.609	.117
Age	−0.05	−0.32 to 0.22	−0.007	0.020	−0.361	.719
Sensory reactivity* cond.	−0.37	−0.73 to −0.01	−4.335	2.074	−2.090	**.046**
*Y* = SWA 58 nap recordings, 35 participants						
Condition (B vs S)	0.28	−0.09 to 0.65	129.65	84.68	1.531	.138
Sensory reactivity	−0.33	−0.62 to −0.03	−381.79	170.35	−2.241	**.032**
Sex (0 = M, 1 = F)	−0.32	−0.92 to 0.27	−148.79	135.95	−1.094	.282
Age	0.03	−0.25 to 0.31	0.422	2.08	0.203	.840
Sensory reactivity* cond.	−0.14	−0.51 to 0.23	−164.85	212.44	−0.776	.445
*Y* = sleep spindle density 57 nap recordings, 35 participants						
Condition (B vs S)	−0.26	−0.74 to 0.23	−0.193	0.183	−1.054	.302
Sensory reactivity	0.23	−0.04 to 0.50	0.436	0.253	1.723	.097
Sex (0 = M, 1 = F)	0.31	−0.24 to 0.85	0.230	0.203	1.135	.266
Age	0.11	−0.16 to 0.38	0.003	0.003	0.802	.428
Sensory reactivity* cond.	−0.67	−1.08 to −0.27	−1.279	0.383	−3.337	**.003**
*Y* = sigma activity 58 nap recordings, 35 participants						
Condition (B vs S)	0.13	−0.36 to 0.63	0.873	1.604	0.544	.590
Sensory reactivity	0.06	−0.22 to 0.34	1.000	2.283	0.438	.664
Sex (0 = M, 1 = F)	0.09	−0.48 to 0.65	0.567	1.829	0.310	.759
Age	0.11	−0.17 to 0.39	0.024	0.030	0.815	.420
Sensory reactivity* Cond	−0.23	−0.72 to 0.26	−3.839	4.007	−0.958	.346
*Y* = K-complex likelihood 29 stimulation naps, 29 participants						
Stim. window (OFF vs ON)	0.38	0.09 to 0.67	0.380	0.150	2.527	**.011**
Sensory reactivity	−0.21	−0.39 to −0.03	−0.557	0.247	−2.259	**.024**
Sex (0 = M, 1 = F)	−0.12	−0.45 to 0.22	−0.118	0.172	−0.688	.491
Age	−0.13	−0.30 to 0.03	−0.004	0.003	−1.522	.128
Sens. reactivity*Stim. window	0.13	−0.24 to 0.49	0.335	0.502	0.666	.505
*Y* = sleep spindle likelihood 29 stimulation naps, 29 participants						
Stim window (OFF vs ON)	−0.05	−0.34 to 0.23	−0.054	0.145	−0.370	.712
Sensory reactivity	0.13	−0.09 to 0.34	0.336	0.295	1.139	.255
Sex (0 = M, 1 = F)	−0.10	−0.62 to 0.42]	−0.101	0.263	−0.385	.701
Age	−0.14	−0.39 to 0.11	−0.004	0.004	−1.069	.285
Sens. reactivity*Stim. window	0.07	−0.18 to 0.31	0.176	0.336	0.525	.600

Abbreviations: CI, confidence interval; SE, standard error; Y, outcome variable. In bold: *p*-values < 0.05.

### Sleep spindle density is decreased by stimulation in highly reactive infants

Similarly to SWs, we analysed sleep spindle density and sigma power (9–16 Hz) to understand whether our results are independent of measurement method and how sensory input interferes with different sleep spindle characteristics. Sleep spindle density was not significantly different between the baseline and stimulation nap (Est. = −0.19, *p* = .302). There was no significant main effect of sensory reactivity on sleep spindle density (Est. = 0.44, *p* = .097). However, when there was stimulation, infants with higher scores on sensory reactivity showed a reduction in sleep spindle density compared to the baseline nap (Est. = −1.28, *p* = .003; see Figure 3, D).

To confirm whether these differences are also reflected in sigma power, we ran an exploratory analysis on sigma power. There was no significant difference in sigma power between the baseline and stimulation nap (Est. = 0.87, *p* = .590), nor was there a significant main effect of sensory reactivity on sigma power (Est. = 1.00, *p* = .664; see Figure 3, C). In contrast to sleep spindle density, there was no significant interaction effect between nap condition and sensory reactivity scores (Est. = −3.84, *p* = .346). The results of both models are summarized in Table 2. Both sleep spindle measures were positively correlated in the baseline naps, although not strongly (*r*_s_ = .457, *p* = .011). See Table S9 for correlations between all the measures at baseline.

### Are the effects of stimulation time-locked to stimulus onset?

To explore whether these decreases in sleep spindle and SW densities in the stimulation nap resulted from an immediate disruptive effect of the stimuli on these events, we compared the likelihood of sleep spindles and slow waves in response to stimulation (stimulus ON window) versus in a control time window before stimulation (stimulus OFF window). Further, we tested whether these evoked responses were related to an infant’s sensory profile. These analyses were performed in the stimulation naps only. If an infant’s sensory profile predicts responses in ON but not OFF windows, this suggests that the previously described differences between stimulation and baseline naps, in infants with high sensory reactivity scores, may have resulted from stimulus-locked interference with spindles and SW generation. In contrast, if sleep spindle and SW occurrence varied with sensory profile across both windows, this would indicate that stimulation disrupted the overall microarchitecture of naps.

A total of 34 stimulation naps were recorded. Five nap recordings were excluded from the analysis due to technical failure during the recording session (*n* = 1), bad-quality Cz channels (*n* = 2), and missing responses on Sensory Profile 2 (*n* = 2), resulting in 29 nap recordings for these analyses.

**Figure 4:**
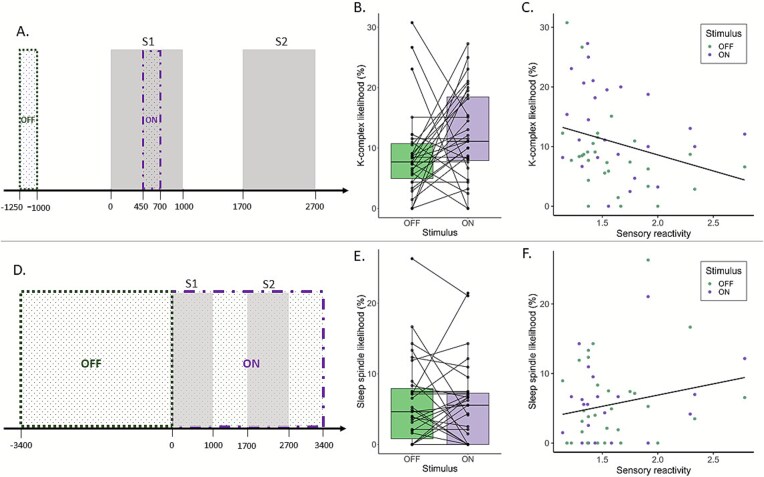
Stimulus OFF and ON methods and results. (A) Detection windows of K-complexes. A K-complex was counted if the peak of a detected K-complex fell in the OFF window (−1250 to −1000 ms relative to S1 onset) or ON window (450–700 ms from S1 onset). (B) Comparison of the likelihood of a K-complex occurring in the ON and OFF windows. Significantly more K-complexes were detected in the ON compared to the OFF windows. (C) K-complex likelihood across both stimulus windows was significantly associated with sensory reactivity scores. There was no significant interaction effect. (D) Detection windows of sleep spindles. A sleep spindle was counted if the start of the sleep spindle was in the OFF (−3400 to 0 ms relative to S1 onset) or ON (0–3400 ms relative to S1 onset). Note that the OFF window contains both S1 and S2. (E) Sleep spindle likelihood in the OFF and ON windows. There was no significant difference between the stimulus windows in sleep spindle likelihood. (F) Sensory reactivity did not significantly predict sleep spindle likelihood across the two stimulus window, nor was there a significant interaction effect between stimulus and sensory reactivity on sleep spindle likelihood.

Sensory stimulation can induce K-complexes—isolated SWs most easily visible in N2—which are thought to index stimulus detection as well as further suppression of that stimulus in an attempt to preserve sleep. In infancy, K-complexes are not easily discernible from the high-amplitude background activity in the EEG signal [71]. This is the first time that we show evoked K-complexes can be automatically detected in infant EEG. K-complex likelihood was measured in windows of 250 ms before and after a stimulus (see Figure 4, A, and more details in the Methods section). The ON window spans 450–700 ms, time-locked to S1. The results show a significant effect of the stimulus window (OFF vs ON) on K-complex likelihood, with more K-complexes in the window after than before S1 (Est. = 0.38, *p* = .011; see Figure 4, B and Table 2). High scores on sensory reactivity also significantly predicted a lower likelihood of a K-complex occurrence, irrespective of the presence or absence of stimulation (Est. = −0.56, *p* = .024; see Figure 4, C). There was no interaction effect of sensory reactivity and the stimulus window (Est. = 0.34, *p* = .505).

Sleep spindle likelihood was measured in a 3400 ms window time-locked to S1 onset (ON window) and a control window of the same length before S1 onset (OFF window; see Figure 4, D). Sleep spindle likelihood did not differ significantly in ON and OFF windows (Est = −0.05, *p* = .712; see Figure 4, E). Sensory reactivity did not have a significant effect on sleep spindle likelihood (Est. = 0.34, *p* = .255; see Figure 4, F), nor did the interaction between sensory reactivity and stimulus windows (Est. = 0.18, *p* = .600).

### Arousals are not affected by stimulation or sensory reactivity

Infants in this sample slept well in the sleep lab with average sleep efficiencies of more than 95% in both nap conditions (see Table 1). Notably, 65% of nap recordings showed no wake periods at all. While infants may not fully wake up, they may have arousals—short and transient periods of increased sensory coupling to the environment, which can be detected behaviorally and in the EEG [72, 73]. Unlike full awakenings, arousals provide a more granular measure of sleep disturbance, especially relevant for naps, which are typically characterized by short or absent wake periods.

As pre-registered, the sleep arrangement (whether the infant slept alone or on the caregiver) was included as a covariate in the mixed model predicting arousal density, but did not have a significant effect on arousals (Est. = 0.023, *p* = .233). This was to account for potential confounds from parental movements that could affect the visual identification of arousals in the EEG signal. There was no significant effect of condition on arousal density (Est. = 0.002, *p* = .837). Neither was there a significant effect of sensory reactivity (Est. = −0.003, *p* = .926), nor was there a significant effect of the interaction between sensory reactivity and condition (Est. = −0.007, *p* = .824). Results are summarized in Table 2.

As a sensitivity analysis, all the models were rerun controlling for autism likelihood (see equation 2). All the results remained similar after autism likelihood status was added (see Table S10).

## Discussion

We found evidence for both associative effects—high sensory reactivity scores associated with reduced sensory decoupling during sleep, regardless of the sensory environment—and a causal role of sensory processing in driving sleep disturbance in highly reactive infants; in the presence of auditory stimulation, infants with high sensory reactivity scores had fewer periods of sensory decoupling, marked by high SW and sleep spindle density.

### Noise or no noise, slow waves are decreased in highly reactive infants

An infant’s sensory profile significantly associates with SWA, both with the power in the SW frequency band and with the number of discrete SWs per minute. Higher sensory reactivity scores predict lower SWA and lower SW densities in a nap, irrespective of the presence or absence of auditory stimulation. Similarly, when looking at K-complexes in sleep stage N2, higher sensory reactivity scores are associated with lower K-complex likelihood, both before and after auditory stimuli. This is in line with one previous study that showed higher self-reported noise sensitivity in nonautistic adults associated with fewer evoked K-complexes to sound [74].

SW activity and/or occurrence are often used as a proxy for homeostatic sleep pressure, as SWA increases after sleep deprivation and dissipates over the course of the night [75–78]. The synaptic homeostasis hypothesis [79] could explain why sensory processing differences and SW properties are associated, unaffected by the sensory environment an infant sleeps in. According to this hypothesis, learning experiences during wakefulness result in an overall increase in synaptic strength and synaptic coupling, which results in large SWs at the start of sleep. The sensory processes underlying high sensory reactivity may interfere with the accumulation of sleep pressure/SWA. Lower SWA in autism mouse models [80] and humans [30]—particularly at the start of the night—suggests the build-up of sleep pressure is weaker in autistic individuals. This could reflect either altered synaptic function and/or potential learning differences, as according to the synaptic homeostasis hypothesis, it is not just neuronal firing that causes an overall increase in synaptic strength but learning specifically [81]. Learning difficulties are common in autism [82, 83] and individuals who feel overwhelmed by sensory input may particularly show disrupted learning [84]. Ineffective learning may result in decreased upscaling of synapses, which may in turn lead to lower SWA at night. Disrupted synaptic plasticity has also been proposed as a potential cause of autism [85, 86], and recent research suggests that synaptic dysfunction may drive both sleep difficulties and other symptoms of autism [87, 88].

The synaptic homeostasis hypothesis also proposes that the decrease of SWA and SWs over the course of sleep reflects the weakening of synapses, essential to facilitate new learning the next day. SWs therefore depend on and drive healthy synaptic functioning. Thus, it is also possible that the association we find between sensory reactivity and SWA results from the fact that reduced SWA, indexing atypical synaptic plasticity during sleep, drives subsequent higher sensory reactivity. In the short term, one night of sleep deprivation has been shown to induce increased reactivity in the somatosensory cortex and decreased sensory gating responses the next day [89, 90]. However, previous work did not assess whether it was SW deprivation that was responsible for these effects, something future studies will have to investigate.

### Auditory input decreases the occurrence of sleep microelements in highly reactive infants

We found that both sleep spindle and SW density were reduced in highly reactive infants during naps with auditory stimulation compared to naps without stimulation. This is not driven by immediate interference from single auditory stimuli since we did not observe a decrease in the likelihood of sleep spindles or K-complexes in highly reactive infants in the poststimulus compared to prestimulus windows. One alternative explanation is that sustained sensory input across a nap may prevent infants with higher sensory reactivity from entering periods of sensory disconnection such as deep sleep. In their comprehensive review on sleep spindles, Fernandez and Lüthi [91] suggest that it is not individual sleep spindles that gate sensory input, but rather periods of increased sleep spindle activity might reflect a period of sensory disconnection. Recently, it has been shown that humans and mice show infraslow fluctuations during sleep of around 50 seconds [92], with phases more and less vulnerable to external perturbation [93, 94]. Periods of less vulnerability are rich in sleep spindles compared to highly vulnerable periods. That we do not find an immediate effect of stimulation on sleep spindle likelihood, but rather a broad influence on sleep spindle density across a nap, may suggest infants with high sensory reactivity are less likely to exhibit these periods of sensory disconnection in the presence of noise.

While we did not observe more arousals in highly reactive infants in the stimulation nap, decreased periods of sensory disconnection may have effects on functions of sleep such as memory consolidation [93, 95].

### Discrepancies between power and density measures

Interestingly, stimulation affected the occurrence of SW or spindle events but not the overall power in the corresponding frequency ranges. One explanation for this is that the activity measures also capture sleep spindles and SWs of lower amplitudes below the detection thresholds of the algorithms. Indeed our study and others show that sleep spindles and sigma activity are not perfectly correlated [59]. Using an alternative method of sleep spindle detection based on time–frequency representation of the signal rather than just time representations, Dimitrov et al. [96] show that while increases in sigma activity often overlap with visually detected sleep spindles, more increases in sigma activity are present in the signal. Another recent study measured the “quality” of a sleep spindle using a metric that captures the decay in amplitude of the sigma oscillation—reflecting its strength and stability—called o-quality [97]. The o-quality of a sleep spindle is related to its distribution across the brain and reflects the degree of network synchronization. The quality (rather than overall amount) of sleep spindles may decrease in infants with high sensory reactivity scores. Low-quality sleep spindles are not coupled as often to slow waves—which is important for effective memory consolidation [98]—and provide less sleep protection [97].

Another explanation is that the discrepancy between density and activity measures reflects the imperfect automated detection of SWs and sleep spindles. As shown for sleep spindles, automated event detection scores in our infant sample were acceptable (*F*_1_ score = 0.55) and similar to other automated detection algorithms in the field [99], but still not as effective as the original A7 algorithm in adults (*F*_1_ = 0.7) [60].

### Infants have shorter naps in noisy environments

The auditory stimulation used in this study (60 dB pure tones) had a minimal effect on sleep macroarchitecture and arousals. We found that sleep duration rather than arousals was most affected by auditory stimulation across all infants, independent of sensory reactivity scores. Using actigraphy at home, Blume et al. [100] also found infants (without an older sibling) had shorter nighttime sleep duration when traffic noise was higher, but not more night awakenings. Therefore, while infants may not seem disturbed by auditory input overtly, their nap may still be significantly shorter in a noisy environment.

### Limitations and considerations for future infant sleep and sensory studies

When measuring behavioural and affective reactivity to sensory input, the age-appropriateness of the tasks and questions is essential. For our sample of 8–11-month-olds, we used the 7–36-month-old age-appropriate version of the Sensory Profile 2 (SP-2 [66]). Notably, a nonapplicable option was added in this second version of the Infant Sensory Toddler Profile. Likely caregivers chose this option when an item was not age-appropriate for their child, but additional qualitative research is needed to confirm this. These could help inform adaptations needed for a third version of the Sensory Profile.

As our aim was to recruit infants with a range of sensory profiles, it is important to consider whether the sensory reactivity scores captured atypical, in particular increased, sensory reactivity. Sensory Profile 2 provides cutoffs for scores below or above the typical range. In our sample, averaged sensory reactivity scores ranged from 1.14 to 2.78. Six infants scored above 1.9, being classified by SP-2 as “more reactive” than the typical range, and two infants scored above 2.5, corresponding to “much more reactive” than expected based on the normal curves. No infant was classified as “less reactive.” Our recruitment strategy therefore successfully captured infants with elevated sensory reactivity.

In the current study, caregivers were encouraged to recreate typical sleep arrangements as much as possible in the lab, as long as it was consistent across visits. Most caregivers opted to hold their baby during the entire nap (29 out of 41), while the remainder of caregivers laid their infant down once asleep. Some infant studies standardize the sleeping arrangement, e.g. all infants sleep in a car seat [52] but these tend to be much younger infants (i.e. 3 month olds in reference [52]), who do not yet have clear sleep arrangements and routines. We therefore aimed to increase ecological validity by preserving infants’ typical routines, but this may have caused slight variations in the perceived loudness of the auditory input. When comparing sleep arrangements (in caregiver’s arms vs alone), we found no effects on sleep microarchitecture, arousals, or sleep duration, but did find infants who were held who had proportionally less N3 and more N1, than those who slept alone. This suggests sleep stage distributions may be affected by differences in sleeping arrangements, while other aspects of sleep are more robust. However, whether standardizing sleeping arrangements outweighs potential effects of altering an infant’s typical sleep remains untested. It is also possible that poor sleepers, likely underrepresented in infant EEG sleep studies, may be unintentionally excluded when only one type of sleeping arrangement is allowed. Balancing these opposing needs for high-quality infant sleep research remains a challenge for infant sleep research.

## Conclusion

Our findings suggest that higher sensory reactivity, an early emerging trait of autism, interferes with the accumulation of sleep pressure, as indexed by decreased SWA during sleep. In addition, auditory input disturbs sleep microstructure in highly reactive infants. Rather than triggering immediate event-related disruptions, auditory input seems to alter microstructure more broadly across longer timescales, reflected by overall reduced sleep spindle and slow wave densities. Thus, we provide new mechanistic understanding by establishing specific links between behavioral and neural phenotypic traits that span typical and atypical development.

## Supplementary Material

Supplemental_materials_final_zsag010

## Data Availability

Two EEG recordings are shared on the Open Science Framework alongside the code for automated sleep spindle detection (osf.io/3ujr4). Other data are available upon request.

## References

[1] Frank MG, Issa NP, Stryker MP. Sleep enhances plasticity in the developing visual cortex. *Neuron.* 2001;30(1):275–287. 10.1016/S0896-6273(01)00279-3 11343661

[2] Li W, Ma L, Yang G, Gan WB. REM sleep selectively prunes and maintains new synapses in development and learning. *Nat Neurosci*. 2017;20(3):427–437. 10.1038/nn.4479 28092659 PMC5535798

[3] Cook F, Conway LJ, Giallo R, Gartland D, Sciberras E, Brown S. Infant sleep and child mental health: a longitudinal investigation. *Arch Dis Child*. 2020;105(7):655–660. 10.1136/archdischild-2019-318014 32152038

[4] Jaramillo V, Schoch SF, Markovic A, et al. An infant sleep electroencephalographic marker of thalamocortical connectivity predicts behavioral outcome in late infancy. *NeuroImage.* 2023;269:119924. 10.1016/j.neuroimage.2023.119924 36739104

[5] Cortese S, Faraone SV, Konofal E, Lecendreux M. Sleep in children with attention-deficit/hyperactivity disorder: meta-analysis of subjective and objective studies. *J Am Acad Child Adolesc Psychiatry*. 2009;48(9):894–908. 10.1097/CHI.0b013e3181ac09c9 19625983

[6] Verhoeff ME, Blanken LME, Kocevska D, et al. The bidirectional association between sleep problems and autism spectrum disorder: a population-based cohort study. *Mol Autism*. 2018;9(1):8. 10.1186/s13229-018-0194-8 29423134 PMC5791216

[7] Kamara D, Beauchaine TP. A review of sleep disturbances among infants and children with neurodevelopmental disorders. *Rev J Autism Dev Disord*. 2020;7(3):278–294. 10.1007/s40489-019-00193-8 33344102 PMC7747783

[8] MacDuffie KE, Shen MD, Dager SR, et al. Sleep onset problems and subcortical development in infants later diagnosed with autism spectrum disorder. *Am J Psychiatry.* 2020;177(6):518–525. 10.1176/appi.ajp.2019.19060666 32375538 PMC7519575

[9] De Laet A, Piccardi ES, Begum-Ali J, et al. Neuronal gating of tactile input and sleep in 10-month-old infants at typical and elevated likelihood for autism spectrum disorder. *Sci Rep*. 2022;12(1):14188. 10.1038/s41598-022-18018-w 35986046 PMC9391390

[10] Begum-Ali J, Gossé LK, Mason L, et al. Infant sleep predicts trajectories of social attention and later autism traits. *J Child Psychol Psychiatry*. 2023;64(8):1200–1211. 10.1111/jcpp.13791 36991307 PMC10952761

[11] Deliens G, Peigneux P. Sleep–behaviour relationship in children with autism spectrum disorder: methodological pitfalls and insights from cognition and sensory processing. *Dev Med Child Neurol*. 2019;61(12):1368–1376. 10.1111/dmcn.14235 30968406

[12] Reynolds S, Lane SJ. Sensory overresponsivity and anxiety in children with ADHD. *Am J Occup Ther*. 2009;63(4):433–440. 10.5014/ajot.63.4.433 19708472

[13] Estes A, Zwaigenbaum L, Gu H, et al. Behavioral, cognitive, and adaptive development in infants with autism spectrum disorder in the first 2 years of life. *J Neurodev Disord*. 2015;7(1):24. 10.1186/s11689-015-9117-6 PMC4511527 26203305

[14] Sacrey LAR, Zwaigenbaum L, Bryson S, et al. Can parents’ concerns predict autism spectrum disorder? A prospective study of high-risk siblings from 6 to 36 months of age. *J Am Acad Child Adolesc Psychiatry*. 2015;54(6):470–478. 10.1016/j.jaac.2015.03.014 26004662

[15] Lane SJ, Leão MA, Spielmann V. Sleep, sensory integration/processing, and autism: a scoping review. *Front Psychol*. 2022;13:877527. https://www.frontiersin.org/articles/10.3389/fpsyg.2022.877527 35656493 10.3389/fpsyg.2022.877527 PMC9152214

[16] Appleyard K, Schaughency E, Taylor B, et al. Sleep and sensory processing in infants and toddlers: a cross-sectional and longitudinal study. *Am J Occup Ther*. 2020;74(6):7406205010p1–7406205010p12. 10.5014/ajot.2020.038182 33275561

[17] Coenen A, Vendrik AJH. Determination of the transfer ratio of cat’s geniculate neurons through quasi-intracellular recordings and the relation with the level of alertness. *Exp Brain Res*. 1972;14(3):227–242. 10.1007/BF00816160 4340696

[18] Coenen A . Sensory gating and gaining in sleep: the balance between the protection of sleep and the safeness of life (a review). *J Sleep Res*. 2024;33(5):e14152. 10.1111/jsr.14152 38286435

[19] Andrillon T, Kouider S. The vigilant sleeper: neural mechanisms of sensory (de)coupling during sleep. *Curr Opin Physiol*. 2020;15:47–59. 10.1016/j.cophys.2019.12.002

[20] Neckelmann D, Ursin R. Sleep stages and EEG power spectrum in relation to acoustical stimulus arousal threshold in the rat. *Sleep.* 1993;16(5):467–477. 10.1093/sleep/16.5.467 8378687

[21] Dang-Vu TT, McKinney SM, Buxton OM, Solet JM, Ellenbogen JM. Spontaneous brain rhythms predict sleep stability in the face of noise. *Curr Biol*. 2010;20(15):R626–R627. 10.1016/j.cub.2010.06.032 20692606

[22] Hayat H, Regev N, Matosevich N, et al. Locus coeruleus norepinephrine activity mediates sensory-evoked awakenings from sleep. *Sci Adv*. 2020;6(15):eaaz4232. 10.1126/sciadv.aaz4232 32285002 PMC7141817

[23] Sato Y, Fukuoka Y, Minamitani H, Honda K. Sensory stimulation triggers spindles during sleep stage 2. *Sleep.* 2007;30(4):511–518. 10.1093/sleep/30.4.511 17520796

[24] Bellesi M, Riedner BA, Garcia-Molina GN, Cirelli C, Tononi G. Enhancement of sleep slow waves: underlying mechanisms and practical consequences. *Front Syst Neurosci*. 2014;8:208. https://www.frontiersin.org/article/10.3389/fnsys.2014.00208 25389394 10.3389/fnsys.2014.00208 PMC4211398

[25] Ameen MS, Heib DPJ, Blume C, Schabus M. The brain selectively tunes to unfamiliar voices during sleep. *J Neurosci*. 2022;42(9):1791–1803. 10.1523/JNEUROSCI.2524-20.2021 35039445 PMC8896625

[26] Chilakamarri P, Thurm A, Farmer C, et al. Characterizing sleep spindles in children with autism spectrum disorder (ASD), developmental delay and neurotypical development. (P3.209). *Neurology*. 2017;88(16_supplement):P3.209. 10.1212/WNL.88.16_supplement.P3.209

[27] Farmer CA, Chilakamarri P, Thurm AE, Swedo SE, Holmes GL, Buckley AW. Spindle activity in young children with autism, developmental delay, or typical development. *Neurology.* 2018;91(2):e112–e122. 10.1212/WNL.0000000000005759 29875224 PMC6053112

[28] Mylonas D, Machado S, Larson O, et al. Dyscoordination of non-rapid eye movement sleep oscillations in autism spectrum disorder. *Sleep.* 2022;45(3). 10.1093/sleep/zsac010 35022792

[29] Tani P, Lindberg N, Nieminen-von Wendt T, et al. Sleep in young adults with Asperger syndrome. *Neuropsychobiology.* 2004;50(2):147–152. 10.1159/000079106 15292669

[30] Arazi A, Meiri G, Danan D, et al. Reduced sleep pressure in young children with autism. *Sleep.* 2020;43(6). 10.1093/sleep/zsz309 31848619

[31] Page J . Characterizing early development and NREM sleep in infants and toddlers and risk for autism disorder. [Master's thesis]. North Carolina, US: University of North Carolina at Chapel Hill; 2018. 10.17615/8m09-6n17

[32] Nguyen J, Zhang B, Hanson E, Mylonas D, Maski K. Neurobehavioral associations with NREM and REM sleep architecture in children with autism spectrum disorder. *Children.* 2022;9(9):1322. 10.3390/children9091322 PMC9497778 36138632

[33] Lambert A, Tessier S, Rochette AC, Scherzer P, Mottron L, Godbout R. Poor sleep affects daytime functioning in typically developing and autistic children not complaining of sleep problems: a questionnaire-based and polysomnographic study. *Res Autism Spectr Disord*. 2016;23:94–106. 10.1016/j.rasd.2015.11.010

[34] Andrillon T, Poulsen AT, Hansen LK, Léger D, Kouider S. Neural markers of responsiveness to the environment in human sleep. *J Neurosci*. 2016;36(24):6583–6596. 10.1523/JNEUROSCI.0902-16.2016 27307244 PMC6601917

[35] Halász P . The K-complex as a special reactive sleep slow wave – a theoretical update. *Sleep Med Rev*. 2016;29:34–40. 10.1016/j.smrv.2015.09.004 26606317

[36] He JL, Williams ZJ, Harris A, et al. A working taxonomy for describing the sensory differences of autism. *Mol Autism*. 2023;14(1):15. 10.1186/s13229-022-00534-1 37041612 PMC10091684

[37] Tavassoli T, Miller LJ, Schoen SA, Nielsen DM, Baron-Cohen S. Sensory over-responsivity in adults with autism spectrum conditions. *Autism.* 2014;18(4):428–432. 10.1177/1362361313477246 24085741

[38] Ben-Sasson A, Gal E, Fluss R, Katz-Zetler N, Cermak SA. Update of a meta-analysis of sensory symptoms in ASD: a new decade of research. *J Autism Dev Disord*. 2019;49(12):4974–4996. 10.1007/s10803-019-04180-0 31501953

[39] Rutter M, Bailey A, Lord C. The Social Communication Questionnaire. Los Angeles: Western Psychological Services; 2003.

[40] Allen C, Silove N, Williams K, Hutchins P. Validity of the social communication questionnaire in assessing risk of autism in preschool children with developmental problems. *J Autism Dev Disord*. 2007;37(7):1272–1278. 10.1007/s10803-006-0279-7 17080270

[41] Wiggins LD, Reynolds A, Rice CE, et al. Using standardized diagnostic instruments to classify children with autism in the study to explore early development. *J Autism Dev Disord*. 2015;45(5):1271–1280. 10.1007/s10803-014-2287-3 25348175 PMC4486213

[42] Franco P, Pardou A, Hassid S, Lurquin P, Groswasser J, Kahn A. Auditory arousal thresholds are higher when infants sleep in the prone position. *J Pediatr*. 1998;132(2):240–243. 10.1016/S0022-3476(98)70438-X 9506634

[43] Trinder J, Newman NM, Le Grande M, et al. Behavioural and EEG responses to auditory stimuli during sleep in newborn infants and in infants aged 3 months. *Biol Psychol*. 1990;31(3):213–227. 10.1016/0301-0511(90)90035-U 2132679

[44] Gramfort A, Luessi M, Larson E, et al. MEG and EEG data analysis with MNE-python. *Front Neurosci*. 2013;7:267. 10.3389/fnins.2013.00267 24431986 PMC3872725

[45] Combrisson E, Vallat R, Eichenlaub JB, et al. Sleep: an open-source Python software for visualization, analysis, and staging of sleep data. *Front Neuroinform*. 2017;11:60. https://www.frontiersin.org/article/10.3389/fninf.2017.00060 28983246 10.3389/fninf.2017.00060 PMC5613192

[46] Combrisson E, Vallat R, O’Reilly C, et al. Visbrain: a multi-purpose GPU-accelerated open-source suite for multimodal brain data visualization. *Front Neuroinform*. 2019;13. 10.3389/fninf.2019.00014 PMC6439346 30967769

[47] Madeleine G-D, David G, Marcus CL, et al. The visual scoring of sleep and arousal in infants and children. *J Clin Sleep Med*. 2007;03(02):201–240. 10.5664/jcsm.26819 17557427

[48] Berry RB, Brooks R, Gamaldo CE, Harding SM, Marcus CL and Vaughn BV for the American Academy of Sleep Medicine. *The AASM Manual for the Scoring of Sleep and Associated Events: Rules, Terminology and Technical Specifications*, Version 2.0. www.aasrnnet.org, Darien, Illinois: American Academy of Sleep Medicine; 2012.

[49] The International Paediatric Work Group on Arousals . The scoring of arousals in healthy term infants (between the ages of 1 and 6 months). *J Sleep Res*. 2005;14(1):37–41. 10.1111/j.1365-2869.2004.00426.x 15743332

[50] Vallat R, Walker MP. An open-source, high-performance tool for automated sleep staging. *eLife*. 2021;10:e70092. 10.7554/eLife.70092 34648426 PMC8516415

[51] Dien J . Issues in the application of the average reference: review, critiques, and recommendations. *Behav Res Methods Instrum Comput*. 1998;30(1):34–43. 10.3758/BF03209414

[52] Sokoloff G, Dooley JC, Glanz RM, et al. Twitches emerge postnatally during quiet sleep in human infants and are synchronized with sleep spindles. *Curr Biol*. 2021;31(15):3426–3432.e4. 10.1016/j.cub.2021.05.038 34139191 PMC8355086

[53] Melendres MC, Marcus CL, Abi-Raad RF, Trescher WH, Lutz JM, Colrain IM. Respiratory-related evoked potentials during sleep in children. *Sleep.* 2008;31(1):55–61. 10.1093/sleep/31.1.55 18220078 PMC2225558

[54] Forget D, Morin CM, Bastien CH. The role of the spontaneous and evoked K-complex in good-sleeper controls and in individuals with insomnia. *Sleep.* 2011;34(9):1251–1260. 10.5665/SLEEP.1250 21886363 PMC3157667

[55] Campbell K . Event-related potentials as a measure of sleep disturbance: a tutorial review. *Noise and Health*. 2010;12(47):137–153. 10.4103/1463-1741.63216 20472959

[56] Louis J, Cannard C, Bastuji H, Challamel MJ. Sleep ontogenesis revisited: a longitudinal 24-hour home polygraphic study on 15 normal infants during the first two years of life. *Sleep.* 1997;20(5):323–333. 10.1093/sleep/20.5.323 9381053

[57] D’Atri A, Novelli L, Ferrara M, Bruni O, De Gennaro L. Different maturational changes of fast and slow sleep spindles in the first four years of life. *Sleep Med*. 2018;42:73–82. 10.1016/j.sleep.2017.11.1138 29458750

[58] Coppieters't Wallant D, Maquet P, Phillips C. Sleep spindles as an electrographic element: description and automatic detection methods. *Neural Plast*. 2016;2016:6783812. 10.1155/2016/6783812 27478649 PMC4958487

[59] Cox R, Schapiro AC, Manoach DS, Stickgold R. Individual differences in frequency and topography of slow and fast sleep spindles. *Front Hum Neurosci*. 2017;11:433. https://www.frontiersin.org/articles/10.3389/fnhum.2017.00433 28928647 10.3389/fnhum.2017.00433 PMC5591792

[60] Lacourse K, Delfrate J, Beaudry J, Peppard P, Warby SC. A sleep spindle detection algorithm that emulates human expert spindle scoring. *J Neurosci Methods*. 2019;316:3–11. 10.1016/j.jneumeth.2018.08.014 30107208 PMC6415669

[61] Warby SC, Wendt SL, Welinder P, et al. Sleep-spindle detection: crowdsourcing and evaluating performance of experts, non-experts and automated methods. *Nat Methods*. 2014;11(4):385–392. 10.1038/nmeth.2855 24562424 PMC3972193

[62] Liu MY, Huang A, Huang NE. Evaluating and improving automatic sleep spindle detection by using multi-objective evolutionary algorithms. *Front Hum Neurosci*. 2017;11:261. https://www.frontiersin.org/journals/human-neuroscience/articles/10.3389/fnhum.2017.00261 28572762 10.3389/fnhum.2017.00261 PMC5435763

[63] Sela Y, Vyazovskiy VV, Cirelli C, Tononi G, Nir Y. Responses in rat core auditory cortex are preserved during sleep spindle oscillations. *Sleep.* 2016;39(5):1069–1082. 10.5665/sleep.5758 26856904 PMC4835306

[64] Rudzik F, Thiesse L, Pieren R, et al. Sleep spindle characteristics and arousability from nighttime transportation noise exposure in healthy young and older individuals. *Sleep.* 2018;41(7). 10.1093/sleep/zsy077 29697833

[65] Chen A, Peter V, Burnham D. Auditory ERP response to successive stimuli in infancy. *PeerJ.* 2016;4:e1580. 10.7717/peerj.1580 26855858 PMC4741073

[66] Dunn W . Sensory Profile 2: User’s Manual. Psych Corporation; San Antonio, Texas, US; 2014.

[67] Manelis-Baram L, Meiri G, Ilan M, et al. Sleep disturbances and sensory sensitivities co-vary in a longitudinal manner in pre-school children with autism spectrum disorders. *J Autism Dev Disord*. 2021;52(2):923–937. 10.1007/s10803-021-04973-2 33835353 PMC8033551

[68] Kuznetsova A, Brockhoff PB, Christensen RHB. lmerTest Package: tests in linear mixed effects models. J Stat Soft 2017;**82**(13). 10.18637/jss.v082.i13

[69] Luke SG . Evaluating significance in linear mixed-effects models in R. Behav Res Methods. 2016;49(4):1494‑1502. 10.3758/s13428-016-0809-y 27620283

[70] Ventura S, Mathieson SR, O’Toole JM, Livingstone V, Ryan MA, Boylan GB. Electroencephalographic sleep macrostructure and sleep spindles in early infancy. *Sleep.* 2022;45(1). 10.1093/sleep/zsab262 PMC8754499 34755881

[71] Metcalf DR, Mondale J, Butler FK. Ontogenesis of spontaneous K-complexes. *Psychophysiology.* 1971;8(3):340–347. 10.1111/j.1469-8986.1971.tb00464.x 5093977

[72] Schieber JP, Muzet A, Ferriere PJ. Les phases d’activation transitoire spontanées au cours du sommeil normal chez l’homme. *Arch Sci Physiol*. 1971;25(4):443–465. 4345798

[73] Halasz P, Kundra O, Rajna P, Pal I, Vargha M. Micro-arousals during nocturnal sleep. *Acta Physiol Acad Sci Hung*. 1979;54(1):1–12. 232612

[74] Lechat B, Hansen K, Micic G, et al. K-complexes are a sensitive marker of noise-related sensory processing during sleep: a pilot study. *Sleep.* 2021;44(9). 10.1093/sleep/zsab065 33710307

[75] Dijk DJ, Beersma DG, Daan S. EEG power density during nap sleep: reflection of an hourglass measuring the duration of prior wakefulness. *J Biol Rhythms*. 1987;2(3):207–219. 10.1177/074873048700200304 2979661

[76] Dijk DJ, Brunner DP, Beersma DGM, Borbély AA. Electroencephalogram power density and slow wave sleep as a function of prior waking and circadian phase. *Sleep.* 1990;13(5):430–440. 10.1093/sleep/13.5.430 2287855

[77] Esser SK, Hill SL, Tononi G. Sleep homeostasis and cortical synchronization: I. Modeling the effects of synaptic strength on sleep slow waves. *Sleep.* 2007;30(12):1617–1630. 10.1093/sleep/30.12.1617 18246972 PMC2276134

[78] Pappenheimer J, Koski G, Fencl V, Karnovsky M, Krueger J. Extraction of sleep-promoting factor S from cerebrospinal fluid and from brains of sleep-deprived animals. *J Neurophysiol*. 1975;38(6):1299–1311. 10.1152/jn.1975.38.6.1299 1221075

[79] Tononi G, Cirelli C. Sleep function and synaptic homeostasis. *Sleep Med Rev*. 2006;10(1):49–62. 10.1016/j.smrv.2005.05.002 16376591

[80] Medina E, Schoch H, Ford K, Wintler T, Singletary KG, Peixoto L. Shank3 influences mammalian sleep development. *J Neurosci Res*. 2022;100(12):2174–2186. 10.1002/jnr.25119 36056598 PMC9588578

[81] Vyazovskiy VV, Walton ME, Peirson SN, Bannerman DM. Sleep homeostasis, habits and habituation. *Curr Opin Neurobiol*. 2017;44:202–211. 10.1016/j.conb.2017.05.002 28575718

[82] Kercood S, Grskovic JA, Banda D, Begeske J. Working memory and autism: a review of literature. *Res Autism Spectr Disord*. 2014;8(10):1316–1332. 10.1016/j.rasd.2014.06.011

[83] Boucher J, Anns S. Memory, learning and language in autism spectrum disorder. *Autism Dev Lang Impair*. 2018;3:2396941517742078. 10.1177/2396941517742078

[84] Keith JM, Jamieson JP, Bennetto L. The influence of noise on autonomic arousal and cognitive performance in adolescents with autism spectrum disorder. *J Autism Dev Disord*. 2019;49(1):113–126. 10.1007/s10803-018-3685-8 30047097 PMC11980704

[85] Bourgeron T . From the genetic architecture to synaptic plasticity in autism spectrum disorder. *Nat Rev Neurosci*. 2015;16(9):551–563. 10.1038/nrn3992 26289574

[86] Guang S, Pang N, Deng X, et al. Synaptopathology involved in autism spectrum disorder. *Front Cell Neurosci*. 2018;12:470. https://www.frontiersin.org/journals/cellular-neuroscience/articles/10.3389/fncel.2018.00470 30627085 10.3389/fncel.2018.00470 PMC6309163

[87] Missig G, McDougle CJ, Carlezon WA. Sleep as a translationally-relevant endpoint in studies of autism spectrum disorder (ASD). *Neuropsychopharmacology.* 2020;45(1):90–103. 10.1038/s41386-019-0409-5 31060044 PMC6879602

[88] Doldur-Balli F, Imamura T, Veatch OJ, et al. Synaptic dysfunction connects autism spectrum disorder and sleep disturbances: a perspective from studies in model organisms. *Sleep Med Rev*. 2022;62:101595. 10.1016/j.smrv.2022.101595 35158305 PMC9064929

[89] Krause AJ, Prather AA, Wager TD, Lindquist MA, Walker MP. The pain of sleep loss: a brain characterization in humans. *J Neurosci*. 2019;39(12):2291–2300. 10.1523/JNEUROSCI.2408-18.2018 30692228 PMC6433768

[90] Zhang Z, Zhong P, Hu F, et al. An excitatory circuit in the perioculomotor midbrain for non-REM sleep control. *Cell.* 2019;177(5):1293–1307.e16. 10.1016/j.cell.2019.03.041 31031008

[91] Fernandez LMJ, Lüthi A. Sleep spindles: mechanisms and functions. *Physiol Rev*. 2020;100(2):805–868. 10.1152/physrev.00042.2018 31804897

[92] Lázár ZI, Dijk DJ, Lázár AS. Infraslow oscillations in human sleep spindle activity. *J Neurosci Methods*. 2019;316:22–34. 10.1016/j.jneumeth.2018.12.002 30571990 PMC6390176

[93] Lecci S, Fernandez LM, Weber FD, et al. Coordinated infraslow neural and cardiac oscillations mark fragility and offline periods in mammalian sleep. *Sci Adv*. 2017;3(2):e1602026. 10.1126/sciadv.1602026 28246641 PMC5298853

[94] Yüzgeç Ö, Prsa M, Zimmermann R, Huber D. Pupil size coupling to cortical states protects the stability of deep sleep via parasympathetic modulation. *Curr Biol*. 2018;28(3):392–400.e3. 10.1016/j.cub.2017.12.049 29358069 PMC5807087

[95] Weber FD, Wang JY, Born J, Inostroza M. Sleep benefits in parallel implicit and explicit measures of episodic memory. *Learn Mem*. 2014;21(4):190–198. 10.1101/lm.033530.113 24634354 PMC3966543

[96] Dimitrov T, He M, Stickgold R, Prerau MJ. Sleep spindles comprise a subset of a broader class of electroencephalogram events. *Sleep.* 2021;44(9). 10.1093/sleep/zsab099 PMC8436142 33857311

[97] Blanco-Duque C, Bond SA, Krone LB, et al. Oscillatory-quality of sleep spindles links brain state with sleep regulation and function. *Sci Adv*. 2024;10(36):eadn6247. 10.1126/sciadv.adn6247 39241075 PMC11378912

[98] Latchoumane CFV, Ngo HVV, Born J, Shin HS. Thalamic spindles promote memory formation during sleep through triple phase-locking of cortical, thalamic, and hippocampal rhythms. *Neuron.* 2017;95(2):424–435.e6. 10.1016/j.neuron.2017.06.025 28689981

[99] Kwon H, Walsh KG, Berja ED, et al. Sleep spindles in the healthy brain from birth through 18 years. *Sleep.* 2023;46(4). 10.1093/sleep/zsad017 PMC10091086 36719044

[100] Blume C, Schoch S, Vienneau D, et al. Association of transportation noise with sleep during the first year of life: a longitudinal study. *Environ Res*. 2021;203:111776. 10.1016/j.envres.2021.111776 34329637

