# Supplementary material for: Sound asleep: sensory decoupling during sleep depends on an infant’s sensory profile
Source: Sleep. 2026 Jan 20;49(4):zsag010. doi: 10.1093/sleep/zsag010 (PMC13089493; doi:10.1093/sleep/zsag010)
Supplement: Supplemental_materials_final_zsag010 [file supplemental_materials_final_zsag010.docx]

# Supplemental materials

# Sound asleep: Sensory decoupling during sleep depends on an infant’s sensory profile

Anna De Laet^1^, Morgan Whitworth^1^, Hope Fincham^1^, Alpar S. Lazar^1^, Rachael Bedford^2^, Teodora Gliga^1^ & the SNOOSE team

^1^ University of East Anglia, Norwich, UK

^2^ Queen Mary University of London, London, UK

Corresponding author:

Teodora Gliga

University of East Anglia, NR4 7TJ, Norwich, UK

[T.Gliga@uea.ac.uk](mailto:T.Gliga@uea.ac.uk)

Table S1 Deviations from the pre-registration

| Pre-registered | Deviation | Reason | Impact of the deviation |
| --- | --- | --- | --- |
| YASA sleep spindle algorithm thresholds were based on the largest correlation between automated and manual detection results using 7 different threshold settings. | Performance evaluation of different threshold settings was elaborated and refined. | The original method used in the pre-registration lacked methodological rigor and its performance could not be evaluated with standard evaluation metrics, such as F1-score, recall and precision. | Likely improved the accuracy of the automated detection method, providing more reliable results. |
| Sleep spindle density will be calculated as number of sleep spindles in sleep stage N2 and N3 combined/ time in N2 and N3. | Sleep spindle density is calculated in N2 only rather than the combination of N2 and N3. | Previous research suggest that infants may have more sleep spindles in N3 than adults. However, our sample showed low numbers of spindles in N3. Combining N2 and N3 would therefore skewed the density measure toward the proportion of time in N2 over N3. | Provided a more accurate representation of sleep spindle density and increased the validity of the measure. |
| Sleep spindles will be measured as sleep spindle density. Slow waves will be measured as SWA. | Inclusion of sigma activity as a measure for sleep spindles and slow wave density for slow waves. | Weak performance of the original automatic sleep spindle detection warrants caution about the robustness of automated detection methods. Other studies also show that sigma activity and discrete spindles measures do not always correlate strongly. | Including multiple measures of the same phenomenon will provide us with a better indication of the robustness of the results as well as provide new insight into the phenomena. |
| Macro-architecture variables, nap duration and sleep stage distribution, are analysed as “preliminary” analyses without any covariates in the model | Covariates and sensory reactivity are added to the models. | These exploratory analyses were conducted out of interest to better understand relationships between macro-architecture and sensory reactivity. | As these analyses were exploratory, they should be interpreted with caution. |
| Mixed models with proportion variables (sleep stage distribution, KC likelihood and sleep spindle likelihood) were either not specified or a GMM with quasi-binomial distribution was specified | A generalised linear mixed effect model with zero-inflated beta regression was specified instead | Quasi-binomial distribution resulted in singular fit due to within person variance close to 0. Chen et al (2017) recommend a GMM with beta regression for proportion data. These models did not show a singular fit. | Likely more reliable results than a |
| We will test whether there is a difference in Wake After Sleep Onset (WASO) between the baseline and stimulation nap | We did not run these analyses, instead we used arousal density | 65% of the recordings did not have wake periods during the nap. | Avoid unreliable conclusions based on a small subset of the sample. |
| The variable sex will only be included to the models as a control variable if there are significant sex differences in the outcome variables indicated by results of t-tests or non-parametric equivalent tests. | Sex was included as a control variable in all models. | While there were no significant sex differences, we decided to err on the side of caution and add sex to all models, as some variables showed marginally significant differences between sexes. | Increased the severity of the analysis. Avoid misleading results due to sex differences. |
| As a sensitivity check, all models will be run without the EL-ASD subjects | Instead of removing EL-ASD participants, ASD likelihood status was added as a control variable as a sensitivity check | If the original approach was taken, sample sizes would be small, potentially producing misleading results. The new approach allows us to control for ASD likelihood status, without whilst maximising sample size | Maintaining statistical power while still controlling for autism related differences. |
| H1: Do sleep spindles and slow waves protect sleep from disruption? To test this we will look at whether sleep spindle density and/or SWA predict arousal density during the stimulation nap over and above arousal density at baseline | We did not address this question in this paper. | More stimuli, time-locked to these oscillations would provide a better answer to this question. | The results of these pre-registered analyses can be found on OSF (osf.io/bv6nh). |

**Influence of the nap time on micro- and macro-architecture**

A generalized LMM with a zero-inflated beta regression and logit link function was run to assess the effects of nap time (morning vs afternoon) on the distribution of sleep stages. The distribution of sleep stages did not differ significantly depending on the time of the nap (Reference category N1; naptimexN2: Est. = -0.19, p = 0.501; naptimexN3: Est. = -0.118, p = 0.675; naptimexREM: Est. = 0.018, p = 0.959).

Linear mixed effect models were run to test for an effect of nap time on other macro-architecture variables, sleep duration and arousal density, and micro-architecture variables, SW activity and density, sleep spindle density and sigma activity. In case of a singular fit, the random effect of subject was removed and a linear regression model was run instead. Results are displayed in Table S2.

*Table S2 Results of linear mixed effect models checking the effect of nap time (0 = morning, 1 = afternoon) on micro- and macro-architecture variables. ° linear regression model without random effect.*

| Outcome variable | Estimate | p-value |
| --- | --- | --- |
| SW density | 1.640 | 0.387 |
| SW activity | 332.3 | 0.074 |
| Sleep spindle density | -0.474 | 0.159 |
| Sigma activity | -1.536 | 0.580 |
| Sleep duration | 9.961 | 0.174 |
| Arousal density | 0.019 | 0.396 |

**Influence of sleep arrangement on micro- and macro-architecture**

A generalized LMM with a zero-inflated beta regression and logit link function was run to assess the effects of sleeping arrangement (alone vs on caregiver) on the distribution of sleep stages. The distribution of sleep stages significantly differed depending on the sleeping arrangement with the proportion of N3 to N1 decreasing when infants slept in their caregiver’s arms compared to alone (Reference category N1; arrangementxN3: Est. = -0.78, p < .001). Similarly, the proportion of REM to N1 was significantly lower in infants who slept on their caregivers vs those who slept alone (arrangementxREM: Est. = -0.81, p = 0.010). There were no significant differences in the proportion of N2 to N1 depending on sleeping arrangement (arrangementxN2: Est. = -0.35, p = 0.135).

Linear mixed effect models were run to test for an effect of sleep arrangement on other macro-architecture variables, sleep duration and arousal density, and micro-architecture variables, SW activity and density, sleep spindle density and sigma activity. In case of a singular fit, the random effect of subject was removed and a linear regression model was run instead. Results are displayed in Table S3.

*Table S3 Results of linear mixed effect models testing the effect of sleeping arrangement (0 = alone vs 1 = on caregiver) on micro- and macro-architecture variables. ° linear regression model without random effect.*

| Outcome variable | Estimate | p-value |
| --- | --- | --- |
| SW density | -1.171 | 0.342 |
| SW activity | -148.3 | 0.224 |
| Sleep spindle density | 0.028 | 0.904 |
| Sigma activity | -1.108 | 0.557 |
| Sleep duration ° | 4.510 | 0.430 |
| Arousal density | -1.685 | 0.252 |

Table S4 descriptive statistics of detected **arousals**. Mean and standard deviation per sleep stage and condition.

|  |  | **all naps** | | **Baseline** | | **Stimulation** | |
| --- | --- | --- | --- | --- | --- | --- | --- |
|  |  | mean | std | mean | std | mean | std |
| All sleep stages | Count | 5.42 | 3.96 | 5.82 | 4.40 | 4.97 | 3.42 |
|  | duration (min) | 0.84 | 0.58 | 0.93 | 0.92 | 0.77 | 0.54 |
|  | Percentage | 1.48 | 0.94 | 1.47 | 0.84 | 1.49 | 1.05 |
| N1 | Count | 1.81 | 2.02 | 2.12 | 2.24 | 1.50 | 1.76 |
|  | duration (min) | 0.23 | 0.28 | 0.26 | 0.29 | 0.19 | 0.26 |
|  | Percentage | 1.96 | 2.13 | 2.15 | 2.25 | 1.77 | 2.02 |
| N2 | Count | 1.71 | 2.10 | 2.05 | 2.45 | 1.35 | 1.65 |
|  | duration (min) | 0.24 | 0.34 | 0.28 | 0.40 | 0.20 | 0.30 |
|  | Percentage | 1.00 | 1.16 | 1.09 | 1.26 | 0.90 | 1.07 |
| N3 | Count | 1.19 | 1.01 | 1.14 | 1.10 | 1.24 | 0.92 |
|  | duration (min) | 0.19 | 0.19 | 0.16 | 0.19 | 0.22 | 0.19 |
|  | Percentage | 1.16 | 1.73 | 0.90 | 1.10 | 1.43 | 2.17 |
| REM | Count | 1.75 | 1.79 | 1.50 | 1.76 | 1.67 | 1.57 |
|  | duration (min) | 0.21 | 0.23 | 0.19 | 0.26 | 0.18 | 0.22 |
|  | Percentage | 3.22 | 4.13 | 3.36 | 4.90 | 3.06 | 3.09 |

Table S5 descriptive statistics of automatically detected **artefacts**. Mean and standard deviation per sleep stage and condition.

|  |  | **all naps** | | **Baseline** | | **Stimulation** | |
| --- | --- | --- | --- | --- | --- | --- | --- |
|  |  | mean | std | mean | std | mean | std |
| All sleep stages | duration (min) | 2.07 | 1.30 | 2.32 | 1.35 | 1.82 | 1.21 |
|  | Percentage | 3.56 | 1.76 | 3.70 | 1.65 | 3.41 | 1.88 |
| N1 | duration (min) | 0.45 | 0.44 | 0.51 | 0.49 | 0.39 | 0.39 |
|  | Percentage | 3.87 | 2.71 | 4.08 | 3.21 | 3.66 | 2.15 |
| N2 | duration (min) | 0.71 | 0.64 | 0.84 | 0.70 | 0.59 | 0.55 |
|  | Percentage | 3.60 | 2.42 | 3.88 | 2.70 | 3.32 | 2.11 |
| N3 | duration (min) | 0.56 | 0.46 | 0.62 | 0.59 | 0.50 | 0.26 |
|  | Percentage | 2.63 | 1.62 | 2.74 | 1.83 | 2.52 | 1.41 |
| REM | duration (min) | 0.48 | 0.48 | 0.53 | 0.54 | 0.42 | 0.40 |
|  | Percentage | 5.16 | 3.18 | 4.90 | 3.18 | 5.48 | 3.24 |

###### **Adaptation of the sleep spindle detection thresholds**

Python scripts and more comprehensive descriptions and results are available on the Open Science Framework (osf.io/3ujr4).

As a ground truth, sleep spindles were visually identified in artefact-free N2 and N3 in channel C4, using the *Sleep* software (Combrisson et al., 2017) by annotating the start and end time of each sleep spindle. Ten initial recordings were scored by two independent scorers (ADL and MW) and compared until agreement was reached. The remaining recordings were scored by one scorer (MW). In a *first round*, 10 ‘exploratory’ algorithms with varying thresholds were run. The thresholds were lowered systematically by 10% increments from the default algorithm (see results on OSF), this makes the thresholds more lenient. During this phase, all three thresholds were lowered equally. The purpose of the initial step was to identify a broad range for further systematic exploration in *round two*, where each threshold could be varied independently to fine-tune the algorithm’s performance. The average F1-score of all recordings in the training set was calculated to obtain one score per algorithm. The best-performing algorithm was chosen based on the highest average F1-score. Round one revealed the best average F1-scores (0.45-0.58) when the thresholds were lowered to 50-90% compared to the default settings. In this range, all thresholds were varied independently in 5% increments. In total, 343 threshold combinations were tested in the second round.

Table S6 Comparison of the performance on the training (n =29) and validation (n = 29) datasets and the default and adjusted threshold settings.

| Dataset | Precision | | Recall | | F1 | |
| --- | --- | --- | --- | --- | --- | --- |
|  | Mean | SD | Mean | SD | Mean | SD |
| Training: Default thresholds | 0.84 | 0.23 | 0.30 | 0.19 | 0.40 | 0.16 |
| Training: adjusted thresholds | 0.64 | 0.17 | 0.63 | 0.19 | 0.59 | 0.14 |
| Validation: adjusted thresholds | 0.62 | 0.19 | 0.54 | 0.24 | 0.55 | 0.19 |

The best performing algorithm in the training datasets had thresholds set to: rms 85% = 1.275, correlation 80%= 0.52 and relative power 65% = 0.13. These threshold settings were used on the validation set and yielded similar performance metrics (see Table S6).

However, precision, recall and F1-scores are dependent on the number of detected spindles, meaning a single false positive will have greater impact in recordings with fewer spindles compared to those with many. To account for this, we also tested whether the best-performing algorithm showed an improvement compared to the default algorithm in the estimated sleep spindle density (N/min), a measure that adjusts for recording length. Sleep spindle density was calculated for the default algorithm, the best-performing adjusted algorithm and manual detection. Pearson correlations between manual detection and both the default and adjusted algorithms were significant (r = 0.61, p < .001; r = 0.73, p < .001, respectively). To determine whether the adjusted algorithm showed a significant improvement, the two overlapping correlations (sharing the manual detection variable) were compared using Fisher’s Z-transformation for dependent groups. This comparison was conducted using the cocor.dep.groups.overlap function from the R package cocor (Diedenhofen & Musch, 2015). The correlation with the adjusted algorithm was significantly higher than the correlation with the default algorithm (z = -1.97, p = 0.049), supporting the choice for the adjusted algorithm (see Fig. 1F). Figure 1E in the main text shows an representative example of an automatically detected sleep spindle with the adjusted thresholds.


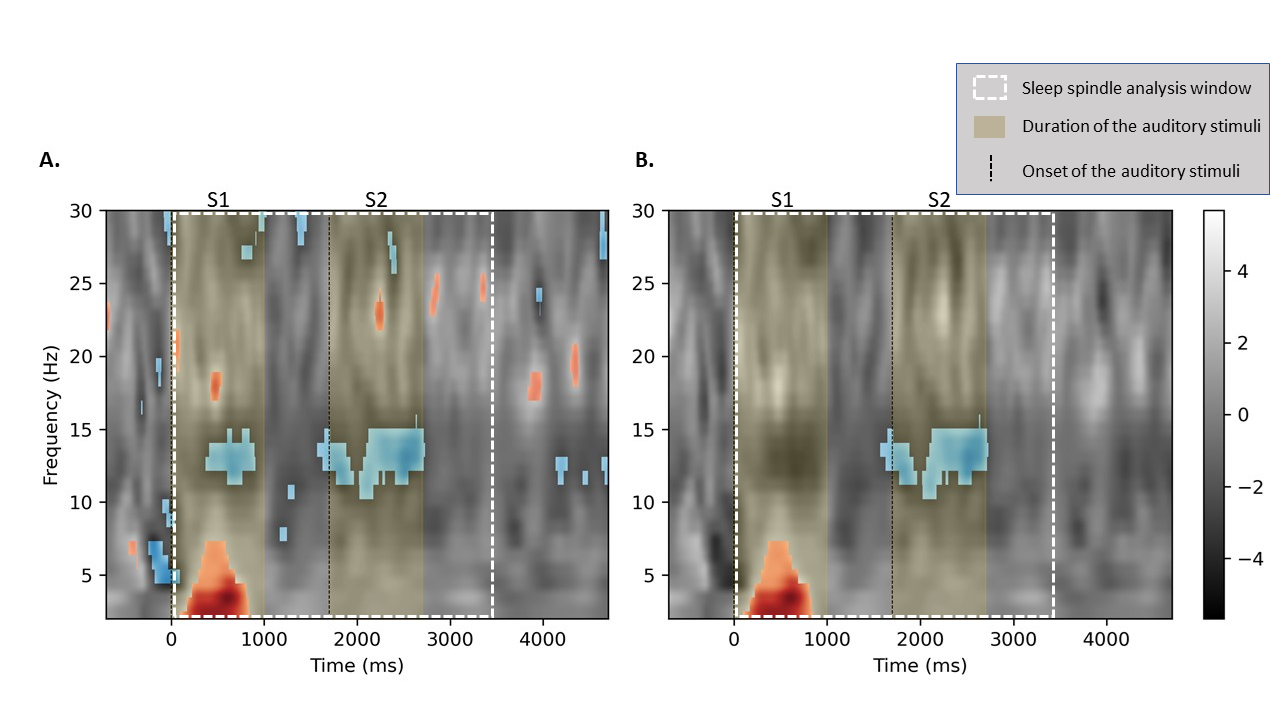


Figure S1 TFR cluster-based permutation. Dashed black vertical lines are the stimulus onsets of S1 and S2. The shaded yellow area is the duration of the stimuli. The white dashed rectangular surface is the analysis window in which sleep spindles were detected (Stimulus ON), which were considered evoked by the stimuli. A window of 3.4 seconds was chosen so both S1 and S2 were included and a post-stimulus window of equal length for both stimuli (700 ms). Time frequency plots show no large undetected clusters of activity outside of this chosen window. Power was calculated using Morlet wavelets in channel Cz. A baseline correction was applied using a log-ratio at 0.5-0.1 s relative to S1 onset. The signal therefore represents a change in power relative to the pre-stimulus window. One-sample t-tests were run on the baselined power and corrected for multiple comparisons using cluster based permutation. **A.** All detected clusters before permutation. **B.** Clusters that have a p-value < 0.1 after permutation.

**Robustness check**

To ensure the results were not affected by the Winsorising of outliers, a robustness check was run by removing the outliers. The same patterns of results and significance emerged.

Table S7 A comparison of the results presented in the manuscript (with two Winsorised values for sleep spindle density and one for arousal density) and results when those outliers were removed entirely.

|  | Winsorised results (β, p) | | Robustness check (β, p) | |
| --- | --- | --- | --- | --- |
| Y = **Sleep spindle density** | |  | |  |
| Condition (B vs S) | | -0.193 (0.302) | | -0.120 (0.489) |
| Sensory reactivity | | 0.436 (0.097) | | 0.169 (0.536) |
| Sex (0=M, 1=F) | | 0.230 (0.266) | | 0.141 (0.496) |
| Age | | 0.003 (0.428) | | 0.003 (0.292) |
| Sensory reactivity* Condition | | **-1.279 (0.003)** | | **-0.992 (0.033)** |
| Y =  **Arousal density** | |  | |  |
| Condition (B vs S) | | 0.002 (0.837) | | 0.008 (0.462) |
| Sensory reactivity | | -0.003 (0.926) | | -0.010 (0.728) |
| Sex (0=M, 1=F) | | 0.001 (0.959) | | -0.003 (0.892) |
| Age | | 0.000 (0.779) | | 0.000 (0.770) |
| Sleep arrangement | | -0.023 (0.234) | | -0.025 (0.171) |

Table S8 results of the linear mixed effect models with sleep spindle duration, sleep spindle power and sleep spindle frequency as outcome variables. SE = standard error.

|  | Estimate | SE | t statistic | p value |
| --- | --- | --- | --- | --- |
| Y = **Sleep spindle duration** |  |  |  |  |
| Condition (B vs S) | 0.006 | 0.025 | 0.248 | 0.806 |
| Sensory reactivity | 0.110 | 0.062 | 1.185 | 0.087 |
| Sex (0=M, 1=F) | 0.061 | 0.049 | 1.147 | 0.221 |
| Age | -0.0002 | 0.0007 | -0.322 | 0.743 |
| Sensory reactivity* Condition | -0.147 | 0.05 | -2.567 | **0.017*** |
| Y =  **Sleep spindle power** |  |  |  |  |
| Condition (B vs S) | 0.083 | 0.069 | 1.193 | 0.241 |
| Sensory reactivity | -0.002 | 0.107 | -0.016 | 0.987 |
| Sex (0=M, 1=F) | 0.128 | 0.085 | 1.512 | 0.139 |
| Age | 0.002 | 0.001 | 1.109 | 0.274 |
| Sensory reactivity* Condition | -0.174 | 0.175 | -0.997 | 0.326 |
| Y =  **Sleep spindle frequency** |  |  |  |  |
| Condition (B vs S) | -0.114 | 0.055 | -2.077 | **0.050*** |
| Sensory reactivity | -0.025 | 0.252 | -0.098 | 0.922 |
| Sex (0=M, 1=F) | 0.403 | 0.196 | 2.051 | **0.048*** |
| Age | -0.001 | 0.002 | -0.601 | 0.5503 |
| Sensory reactivity* Condition | 0.0 | 0.143 | 0.594 | 0.559 |

Table S9 Spearman correlations between the main variables in the baseline nap. * p <0.05 - >0.01, **p <0.01 - >0.001, *** p<0.001

| Variable | 1 | 2 | 3 | 4 | 5 | 6 | 7 | 8 |
| --- | --- | --- | --- | --- | --- | --- | --- | --- |
| 1. Sensory reactivity | - |  |  |  |  |  |  |  |
| 2. Age | .143 | - |  |  |  |  |  |  |
| 3. Nap duration | -.101 | .052 | - |  |  |  |  |  |
| 4. Arousal density | .015 | -.136 | .163 | - |  |  |  |  |
| 5. Sigma activity | -.007 | -.101 | **-.509**** | -.244 | - |  |  |  |
| 6. Sleep spindle density | **.406*** | .042 | -.151 | -.101 | **.457*** | - |  |  |
| 7. SWA | **-.451*** | -.266 | -.343 | -.259 | .144 | -.325 | - |  |
| 8. SW density | -.302 | -.197 | -.277 | -.115 | .353 | .169 | **.677***** | - |

Table S10 Summary of the results with and without autism likelihood as a covariate.

|  | **Standard model** | | **Model + autism likelihood** | |
| --- | --- | --- | --- | --- |
|  | Estimate | p value | Estimate | p value |
| Y = **nap duration** |  |  |  |  |
| Condition (B vs S) | -12.125 | 0.018 | -12.214 | **0.016** |
| Sensory reactivity | 0.453 | 0.948 | -0.604 | 0.931 |
| Sex (0=M, 1=F) | 0.428 | 0.940 | 0.932 | 0.871 |
| Age | 0.047 | 0.595 | 0.039 | 0.661 |
| Autism likelihood (TL vs EL) | / | / | 6.091 | 0.369 |
| Sensory reactivity* Condition | 6.158 | 0.621 | 5.875 | 0.634 |
| Y = **arousal density** |  |  |  |  |
| Condition (B vs S) | 0.002 | 0.837 | 0.002 | 0.836 |
| Sensory reactivity | -0.003 | 0.926 | 0.003 | 0.913 |
| Sex (0=M, 1=F) | 0.001 | 0.959 | -0.002 | 0.936 |
| Age | 0.000 | 0.779 | 0.000 | 0.844 |
| Sleep arrangement | -0.023 | 0.234 | -0.022 | 0.254 |
| Autism likelihood (TL vs EL) | / | / | -0.024 | 0.344 |
| Sensory reactivity* Condition | -0.007 | 0.824 | -0.008 | 0.789 |
| Y = **SW density** |  |  |  |  |
| Condition (B vs S) | -0.473 | 0.598 | -0.481 | 0.591 |
| Sensory reactivity | -3.672 | **0.033** | -3.787 | **0.030** |
| Sex (0=M, 1=F) | -2.118 | 0.117 | -2.082 | 0.124 |
| Age | -0.007 | 0.719 | -0.007 | 0.722 |
| Autism likelihood (TL vs EL) | / | / | 0.574 | 0.713 |
| Sensory reactivity* Condition | -4.335 | **0.046** | -4.339 | **0.045** |
| Y = **SWA** |  |  |  |  |
| Condition (B vs S) | 129.65 | 0.138 | 130.13 | 0.138 |
| Sensory reactivity | -381.79 | **0.032** | -364.28 | **0.042** |
| Sex (0=M, 1=F) | -148.79 | 0.282 | -153.83 | 0.264 |
| Age | 0.422 | 0.840 | 0.40 | 0.847 |
| Autism likelihood (TL vs EL) | / | / | -87.97 | 0.583 |
| Sensory reactivity* Condition | -164.85 | 0.445 | -164.609 | 0.447 |
| Y = **Sleep spindle density** |  |  |  |  |
| Condition (B vs S) | -0.193 | 0.302 | -0.191 | 0.305 |
| Sensory reactivity | 0.436 | 0.097 | 0.445 | 0.096 |
| Sex (0=M, 1=F) | 0.230 | 0.266 | 0.228 | 0.273 |
| Age | 0.003 | 0.428 | 0.003 | 0.418 |
| Autism likelihood (TL vs EL) | / | / | -0.049 | 0.837 |
| Sensory reactivity* Condition | -1.279 | **0.003** | -1.280 | 0.003 |
| Y = **Sigma activity** |  |  |  |  |
| Condition (B vs S) | 0.873 | 0.590 | 0.811 | 0.610 |
| Sensory reactivity | 1.000 | 0.664 | 0.133 | 0.952 |
| Sex (0=M, 1=F) | 0.567 | 0.759 | 0.844 | 0.627 |
| Age | 0.024 | 0.420 | 0.021 | 0.457 |
| Autism likelihood (TL vs EL) | / | / | 4.547 | **0.031** |
| Sensory reactivity* Condition | -3.839 | 0.346 | -3.784 | 0.345 |
| Y = **K-complex likelihood** |  |  |  |  |
| Stimulus window (OFF vs ON) | 0.380 | **0.011** | 0.380 | 0.012 |
| Sensory reactivity | -0.557 | **0.024** | -0.556 | 0.024 |
| Sex (0=M, 1=F) | -0.118 | 0.491 | -0.116 | 0.510 |
| Age | -0.004 | 0.128 | -0.004 | 0.129 |
| Autism likelihood (TL vs EL) | / | / | 0.016 | 0.930 |
| Sensory reactivity*Stimulus window | 0.335 | 0.505 | 0.333 | 0.509 |
| Y = **sleep spindle likelihood** |  |  |  |  |
| Stimulus window (OFF vs ON) | -0.054 | 0.712 | -0.058 | 0.690 |
| Sensory reactivity | 0.336 | 0.255 | 0.316 | 0.294 |
| Sex (0=M, 1=F) | -0.101 | 0.701 | -0.103 | 0.695 |
| Age | -0.004 | 0.285 | -0.005 | 0.266 |
| Autism likelihood (TL vs EL) | / | / | 0.095 | 0.762 |
| Sensory reactivity*Stimulus window | 0.176 | 0.600 | 0.189 | 0.575 |
